# Cu^2+^ coordination-induced in situ photo-to-heat on catalytic sites to hydrolyze β-lactam antibiotics pollutants in waters

**DOI:** 10.1073/pnas.2302761120

**Published:** 2023-12-18

**Authors:** Jiazhen Li, Dongge Ma, Qiang Huang, Yangyang Du, Qin He, Hongwei Ji, Wanhong Ma, Jincai Zhao

**Affiliations:** ^a^Key Laboratory of Photochemistry, Institute of Chemistry, Chinese Academy of Sciences, Beijing 100190, People’s Republic of China; ^b^Beijing National Laboratory for Molecular Sciences, Beijing 100190, People’s Republic of China; ^c^School of Chemical Sciences, University of Chinese Academy of Sciences, Beijing 100049, People’s Republic of China; ^d^Department of Chemistry, College of Chemistry and Materials Engineering, Beijing Technology and Business University, Beijing 100048, People’s Republic of China

**Keywords:** β-lactam antibiotics hydrolysis, photothermal conversion effect, Cu^2+^, COFs

## Abstract

For degradation of β-lactam antibiotics pollution in waters, the strained β-lactam ring is the most toxic and resistant moiety to biodegrade and redox-chemically treat among their functional groups. Hydrolytically opening β-lactam ring with Lewis acid catalysts has long been recognized as a shortcut, but at room temperature, such hydrolysis is too slow to be deployed. Here, we found when Cu^2+^ was immobilized on imine-linked COF (covalent organic framework) (Cu^2+^/Py-Bpy-COF, Cu^2+^ load is 1.43 wt%), as-prepared composite can utilize the light irradiation (wavelength range simulated sunlight) to in situ heat anchored Cu^2+^ Lewis acid sites through an excellent photothermal conversion to open the β-lactam ring followed by a desired full-decarboxylation of hydrolysates. Under 1 W/cm^2^ simulated sunlight, Cu^2+^/Py-Bpy-COF powders placed in a microfiltration membrane rapidly cause a temperature rising even to ~211.7 °C in 1 min. It can effectively hydrolyze common β-lactam antibiotics in waters and even antibiotics concentration is as high as 1 mM and it takes less than 10 min. Such photo-heating hydrolysis rate is ~24 times as high as under dark and ~2 times as high as Cu^2+^ homogenous catalysis. Our strategy significantly decreases the interference from generally coexisting common organics in waters and potential toxicity concerns of residual carboxyl groups in hydrolysates and opens up an accessible way for the settlement of β-lactam antibiotics pollutants by the only energy source available, the sunlight.

## Significance

Cu^2+^ as hydrolysis catalysts to open the strained β-lactam ring is the closest to being used to the actual treatment of β-lactam antibiotics contamination in waters so far. It is well known that such hydrolysis is sensitive to temperature and is very slow at room temperature, but there has been a lack of a viable means of heating for fast hydrolysis of these ppm-level antibiotics without heating thousands of tons of sewage bodies. Here, we reported an alternative to anchoring Cu^2+^ ion on a covalent organic framework support that is strongly colored and perfectly capable of heating itself (even to ~211.7 °C) through its excellent photothermal conversion of the sunlight irradiation to power the hydrolysis/decarboxylation of target objects.

**Fig. 1.**
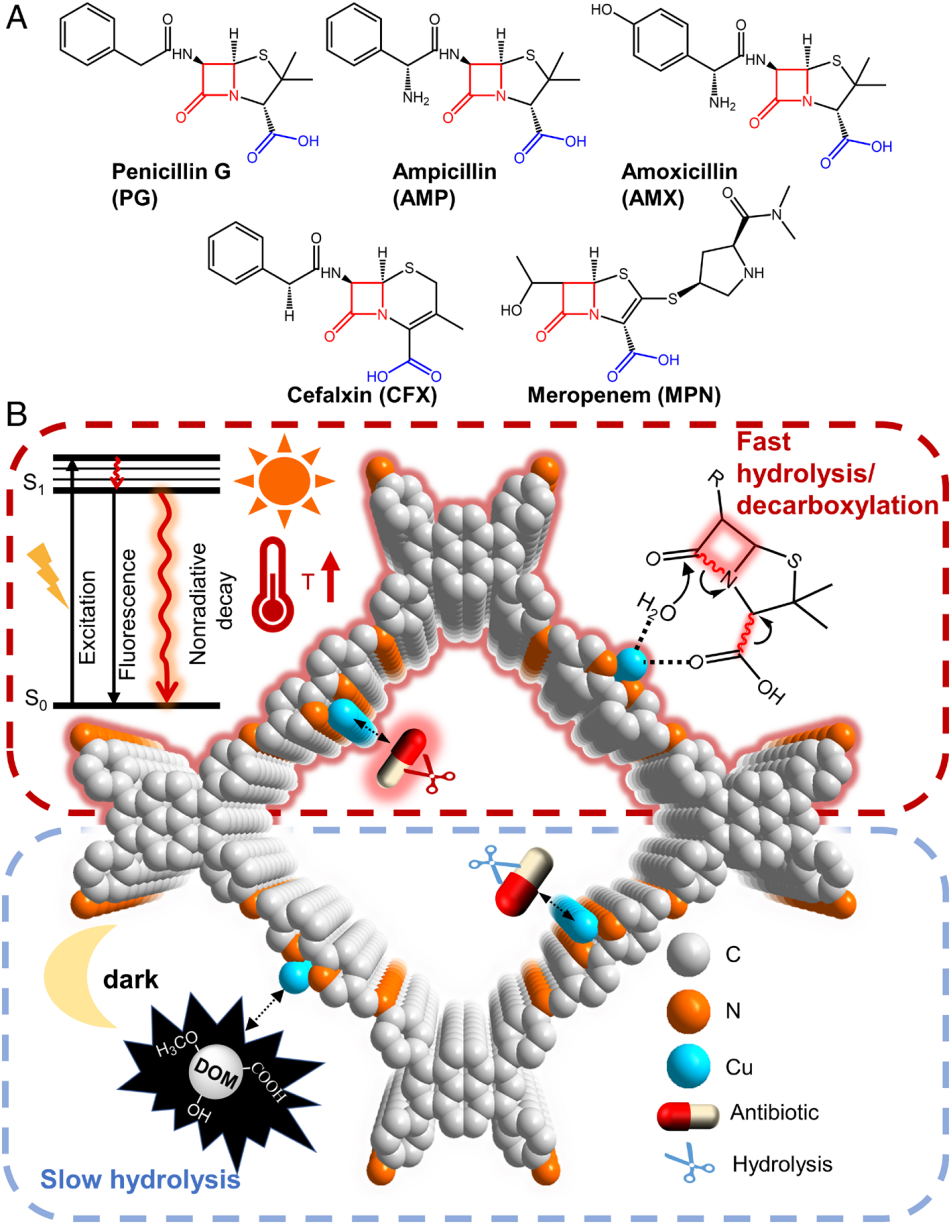
(*A*) Structures of common β-lactam antibiotics. (*B*) Schematic representation of Cu^2+^/COF photothermal hydrolysis/decarboxylation of β-lactam antibiotics.

Antibiotics are a group of important antibacterial agents for fighting bacterial infections and are widely used in medicine, agriculture, animal husbandry, and so on. However, the increasing use of antibiotics all over the world causes significant concerns such as extensive residue in water and soil (1, 2) and the dissemination of antibiotic-resistance genes (3). They have been detected in ng/L to mg/L in wastewater around the world (4) posing a threat to human health (5, 6). There are several major types of common antibiotics such as β-lactam, aminoglycosides, tetracyclines, macrolides, etc. (7). The most prevalently utilized antibiotics are β-lactam antibiotics, accounting for 50 to 70% of total antibiotics consumption (8, 9). Fig. 1*A* shows several common β-lactam antibiotics. Because of their high water-solubility, hydrophilicity, and inherent antimicrobial effects (10, 11), the common biodegradation toward these β-lactam antibiotics in water matrices is nearly invalid (12), furthermore, the active microflora systems are constantly crippled by these antibiotics (13–15). Advanced oxidation technology (AOT) such as photocatalysis (16, 17) and Fenton/photo-Fenton (18) have been identified to have the capacity to treat these antimicrobial pollutants in waters via generating strong oxidizing reactive oxygen species (ROS) such as •OH, •OOH, and ^1^O_2_. However, ROS doesn't have selectivity and is always captured by coexisting dissolved natural organic matter (DOM) in general wastewater and artificial organic pollutants in industrial wastewater (19). In particular, the concentration of these coexisting organics commonly is around mg/L (20) and approximately 1 to 10^3^ times as much as aimed antibiotics, which will rapidly and overwhelmingly exhaust these high-cost ROS before aimed antibiotics degradation according to the collision reaction probability. Besides, some oxidative intermediate products through ROS attacking such as the most common hydroxylation and carboxylation of substrates are generally uncontrollable and may be more toxic than pristine antibiotics (21). Therefore, improving degradation selectivity and decreasing the toxicity of target products are worth the expectation for the actual antibiotics-contaminated water treatments.

The active function moiety of β-lactam antibiotics that prevents common biodegradation is mainly their sharing β-lactam ring (22). Once the β-lactam ring is broken, the opened-ring antibiotic skeleton lacks the bactericidal ability and is readily metabolized and degraded by the common biological treatment (23). Thus, selectively opening the β-lactam ring of the β-lactam antibiotics without the use of these nonselective ROS will be the prospective and feasible strategy, especially in the presence of a lot of other organic matters. At its most straightforward, the strained β-lactam ring can be cleaved by hydrolysis, but its hydrolysis without adding any catalyst is too slow to be applied in wastewater treatment (k ~ 9.58 × 10^−5^ min^−1^, for Penicillin G, at pH7 and 25 °C) (24). A few of research studies focused on degrading β-lactam antibiotics by hydrolysis (25–27) and revealed that such hydrolysis could be accelerated by very small amounts of catalysts such as acid or alkali and metal ions (28). For instance, when the solution pH is adjusted to 2, the hydrolysis rate can be improved by 3 orders (k ~ 0.22 min^−1^, for PG, at pH2 and 25 °C). The promoted degradation of PG in waters in the presence of metal ions such as Zn^2+^ (29), Cu^2+^, Ni^2+^, and Co^2+^ has also been studied by which PG was hydrolyzed along the typical Lewis acid catalytic mechanism (30). Among these Lewis acid catalysts, Cu^2+^ performs the best catalytic effect being able to improve degradation rate by 8 orders in pH2 solution because it has a dual function of hydrolysis and oxidation (31). Nevertheless, there are still major drawbacks to adding these transition metal ions into the actual water treatment systems, e.g., easy to cause secondary pollution; difficult to recycle these ions. Especially, the formed hydrolysates inevitably carry carboxyl groups that commonly possess potential biotoxicity or bio-sensitivity and make the subsequent biodegradation difficult (32).

In addition, as hydrolysis is usually endothermic dominant, it is the most sensitive to reaction temperature. The rate constant increases at an exponential function with temperature increase; namely, it generally improves 2.5 to 3.9-fold for a 10 °C increase in temperature (33). Accordingly, at least two methods would highly benefit the actual treatments of the bulky wastewater containing β-lactam antibiotics pollutants. One is anchoring metal ions on support to recycle; the other is heating the anchored catalytic sites by some available heat sources and tactics to readily conduct heat. For the former, there are some successful attempts for example adsorbed Zn^2+^ on goethite (34). For the latter, heating the bulk wastewater is energy-intensive (35). The photothermal conversion effect may offer the potential to solve this problem in which solar irradiation can be used as an energy source and as-formed heat is readily directed to the catalytic sites instead of heating the whole solution. Especially, the source treatment of the antibiotic manufacturing wastewater by efficiently catalytic hydrolysis, of course, would have better processing efficiency and economic benefits. Very recently, a pioneer work was reported that encapsulating Prussian blue as a photothermal conversion center into ZIF-8 to heat the bio-enzyme catalyzed hydrolysis of cephalosporin under near-infrared laser irradiation (36). It was severely limited to employ bioenzymes acted as the component of the composite catalysts for hydrolyzing antibiotics through such a photothermal strategy because bioenzymes generally do not tolerate too high temperatures. However, the underlying design idea is encouraging. Inspired by this, we propose that a heterogeneously catalytic material that combined a photo-to-heat conversion unit with a fixed-Lewis acid catalyst such as Cu^2+^ ion into one may be solar-powered to realize the effective hydrolysis of β-lactam antibiotic pollutants in most common wastewaters. Hereinto, the structure that both immobilizes Cu^2+^ and creates a photothermal effect on Cu^2+^ sites within the body is the core part of this assembly. Here, we screen out covalent organic frameworks (COFs) as this core function material based on their possessing optional solar light harvest and charge separation efficiency after being complexed with Cu^2+^ that can significantly enhance the photothermal conversion effect for acceleration of Cu^2+^ sites-catalyzed hydrolysis reaction of β-lactam antibiotics, more based on our finding that the combination of COF with Cu^2+^ or Zn^2+^ can add the unique catalytic feature of complete decarboxylation of hydrolysates in parallel with main hydrolysis reaction.

COFs are a class of crystalline porous materials with bi- or three-dimensional structures constructed from organic precursors by dynamic covalent bonds (37). In particular, 2D COFs integrate organic building blocks into covalent 2D sheets and layered frameworks forming directional open nanochannels (38) which are beneficial for the transport, especially larger molecules like antibiotics. COFs as support to immobilize active metal sites for catalysis have been researched widely in recent years (39–43). What is even more profound is that owing to a large π-electronic conjugation and strong π–π interaction between the interlayers, both of which might allow COFs to behave as dipole antennae within broad absorption even to the visible region and release both electron excitation and vibrational energy to produce localized heat (44). Recently, several works have reported COFs’ application for significant photothermal conversion effects even under visible-light irradiation (45–48). This may provide us with an alternative for economically performing hydrolysis of β-lactam antibiotics with the use of sunlight irradiation. However, what structure of COF materials impregnated with active Cu^2+^ sites can possess both significant photothermal conversion effect to directly activate the Cu^2+^ sites before the heat diffuses into the bulky water and the outstanding photo-thermally induced charge separation capacity for full-decarboxylation of hydrolysates accompanied by main hydrolysis of β-lactam antibiotics has ever rarely been specifically designed and tried yet.

Herein, we screen out Bpy [(2,2′-bipyridine)-5,5′-dicarbaldehyde] as a linker to condensation with Py (4,4′,4″,4‴-(Pyrene-1,3,6,8-tetrayl) tetraaniline) to synthesize Py-Bpy-COF (noted as COF) construction, aimed to leave regular N, N′ sites along the COFs’ pores for coordination of Cu^2+^ ions (Fig. 1*B*). As-prepared Cu^2+^/Py-Bpy-COF (noted as Cu^2+^/COF) is stable in water and light irradiation/heating conditions. Cu^2+^ and COF alone have little photothermal conversion effect under the sunlight wavelength range. However, once Cu^2+^ is coordinated into Cu^2+^/COF, its outstanding charge separation state between Cu^2+^ sites and COF support excited by visible light was very in favor of generating photothermal conversion effect to efficiently drive the catalytic hydrolysis of the β-lactam ring of antibiotics and full-decarboxylation of hydrolysates simultaneously. It can effectively hydrolyze common β-lactam antibiotics in waters and it takes less than 10 min even if the concentration of the antibiotic is as high as 1 mM. In different from traditional Cu^2+^-based hydrolysis, as generated main hydrolysates no longer contain carboxyl groups. Thus, the most troublesome coexisted other organic interferences in wastewater treatment for other ROS-based AOT degradation, and potential toxicity concerns of residual carboxyl groups of hydrolysates in hydrolysis degradation have been drastically reduced by our photothermal catalytic hydrolysis strategy.

## Results and Discussion

### Photothermal Performance of Cu^2+^/COF Composite.

By loading different contents of Cu^2+^ to COF, Cu^2+^/COF was obtained and confirmed by IR, XRD (*SI Appendix*, Fig. S2), and ICP (*SI Appendix*, Table S2). The molar ratio of Cu^2+^ to 2,2′-Bpy-DCA unit in COF was not more than 0.7 (~8 wt%), while that of Cu theoretical full loading on Bpy units was 1 (~12 wt%). Such complexed Cu^2+^ sites were relatively stable in water since the ability of coordination between Cu^2+^ and dipyridyl N is strong and coordination constant K is about ~10^8.1^ (49). We chose Cu^2+^/COF (4 wt%) as a representation to conduct characteristics including SEM, HRTEM, Brunauer–Emmett–Teller, thermogravimetric analysis, XPS, X-ray absorption spectroscopy (XANES and EXAFS), and solid-state NMR. The detailed description was shown in *SI Appendix*, Fig. S3 and *Text S7*. We further analyzed their energy band structure (*SI Appendix*, *Text S8*) by Tauc plot, Mott-Schottky, and Valence-band XPS (*SI Appendix*, Fig. S4). In brief, the morphology, surface area, pore size, and thermal stability of COF little changed after loading Cu^2+^, proving the crystalline porous structure of COF remained. The Cu^2+^ site was atomically dispersed and coordinated with four N/O atoms of COF.

**Fig. 2.**
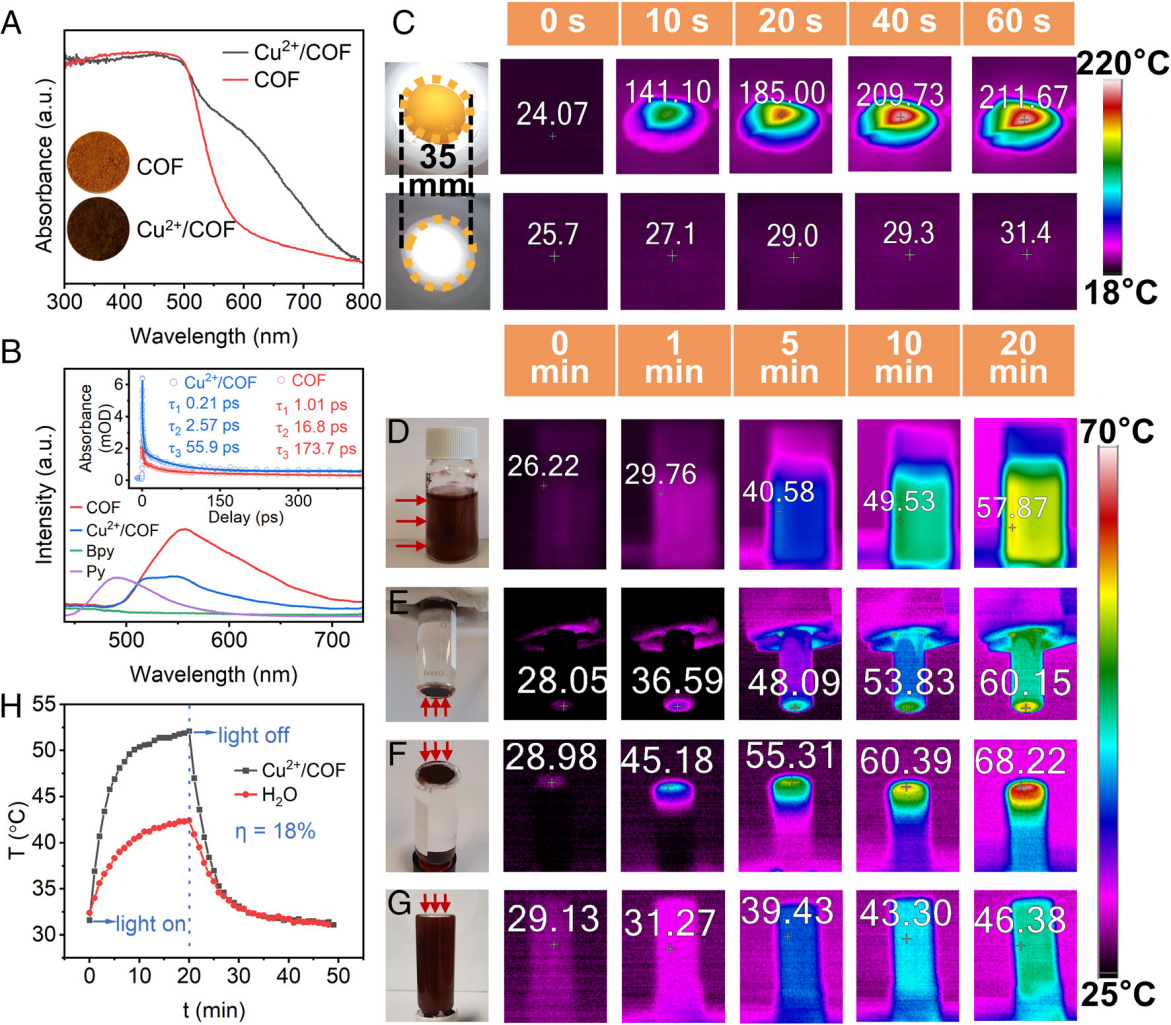
(*A*) UV−vis diffuse reflectance spectra of COF and Cu^2+^/COF (4 wt%) powder. The *Insets* are their powder pellet photographs. (*B*) The fluorescence spectra of Py, Bpy, COF, and Cu^2+^/COF. *Inset*: the kinetic curves and fitting results of femtosecond TA spectra pumped at 375 nm for COF (probed at 675 nm) and Cu^2+^/COF (probed at 700 nm). (*C*) Infrared thermal images of dry Cu^2+^/COF powder deposited on microfiltration membrane (FM, 35 mm) and blank FM under top irradiation, (*D*) Cu^2+^/COF dispersion (0.2 g/L) under stirring and side irradiation. Thermal images when Cu^2+^/COF powders (2 mg) gathered by FM (10 mm) were placed at the (*E*) *Bottom* and (*F*) *Top* of the liquid with bottom and above irradiation respectively. (*G*) Thermal images of Cu^2+^/COF dispersion under the same experiment condition with (*F*), (*H*) heating curve under irradiation (illumination area was 1 cm^2^) and cooling curve after turning off the light source of Cu^2+^/COF dispersion (0.2 g/L) and water. The above light power was all 1 W/cm^2^.

In addition, we researched the photophysical properties of Cu^2+^/COF and COF (Details in *SI Appendix*, *Text S9*). As expected, the absorption spectrum of COF was broadened obviously to the visible-light region after loading Cu^2+^ (Fig. 2*A*), which was beneficial to utilize more solar irradiation for promoting photothermal conversion. The fluorescence of COF (at 555 nm) was red-shifted and enhanced compared with its monomer Py (at 490 nm) due to intramolecular charge transfer from Py to Bpy in the excited state (50) (Fig. 2*B*). The fluorescence of COF bulk was significantly quenched and blue-shifted after loading Cu^2+^, causing dual emissions at 518 nm and 550 nm, respectively. The quenching of fluorescence indicated that the absorbed incident light was converted into heat, but this was not caused by the single excited state relaxation. Furthermore, femtosecond transient absorption (TA) spectra (*SI Appendix*, Fig. S5-1 *E* and *F*) were conducted to investigate the ultrafast nonradiative transition (51, 52). The kinetic decay curves and fitting results at maximum absorption wavelength for COF (675 nm) and Cu^2+^/COF (700 nm) are shown in Fig. 2 *B*, *Inset*. Cu^2+^/COF went through three relaxation processes, and their lifetimes were all significantly shorter than that of COF (53), clearly indicating Cu^2+^/COF was beneficial to photothermal conversion.

To see the photothermal conversion effect in action, thermal images were recorded by an infrared thermal camera (The digital photo of the infrared thermal camera was shown in *SI Appendix*, Fig. S6). Under 1 W/cm^2^ simulated sunlight illumination (generated by a 300 W Xe lamp with an AM1.5 filter), Cu^2+^/COF (4 wt%) powder deposited on a microfiltration membrane (FM, 0.22 µm, diameter 35 mm) can be heated significantly from room temperature even to ~211.7 °C in 1 min, while the temperature change of blank FM without Cu^2+^/COF is no more than 5 °C (Fig. 2*C*). In the actual aqueous degradation system, the dispersion of Cu^2+^/COF powder (0.2 g/L) is heated up from room temperature to 58 °C for 20 min under stirring and illumination (Fig. 2*D*). In comparison to the dry condition of Cu^2+^/COF powder on FM, most of the heat created from the photothermal effect was transferred to the surrounding water media. It was mechanical stirring that enforced the heat loss from the Cu^2+^/COF heat source. Thus, we inferred if Cu^2+^/COF powders (2 mg) gathered by FM (diameter 10 mm) were deliberately deposited at the bottom of the vessel and not stirred, the heat produced from the photothermal conversion effect would remarkably focus on the solid Cu^2+^/COF powders as well as limited adsorbed water layers around their boundary. As Fig. 2*E* shows, there was an obvious temperature difference between Cu^2+^/COF and bulk water. The bulk water temperature slightly increased because the density of adsorbed water decreased as its temperature increased which caused heat inevitably to be transferred by bottom-up convection. To verify this reason, Cu^2+^/COF FM (10 mm) was placed on top of the liquid and illuminated from above (Fig. 2*F*). The temperature of Cu^2+^/COF rose from room temperature to 68 °C in 20 min. Besides, the local photo-to-heat effect and heating rate were more significant than that of irradiated from the bottom because the heat diffusion caused by the density change of adsorbed water was inhibited. Although the temperature increase of Cu^2+^/COF dispersion under light was much less than that of dry solid powders, the temperature of Cu^2+^/COF without agitation is 22 °C higher than that with agitation under the same experimental conditions (Fig. 2*G*). This operation is of great significance for the practical application of Cu^2+^/COF with good adiabaticity. Namely, Cu^2+^/COF can accumulate heat on limited catalytic sites to effectively react with aimed pollutant molecules diffused on the surface of the catalyst rather than heat dissipation to bulk water. Finally, to quantificationally evaluate this material’s photothermal conversion efficiency in the practice water system, we recorded Cu^2+^/COF dispersion’s heating curve under irradiation and cooling curve after turning off the light source (Fig. 2*H*). The measured photothermal conversion efficiency η was 18% according to the linear time data versus −lnθ (θ represents the ratio of ΔT to ΔT_max_) of dispersion and water, respectively (*SI Appendix*, Fig. S7), which was better than the commercial photothermal agent indocyanine green (15.1%) (54).

### Photothermally Enhanced Catalytic Degradation of β-Lactam Antibiotics.

**Fig. 3.**
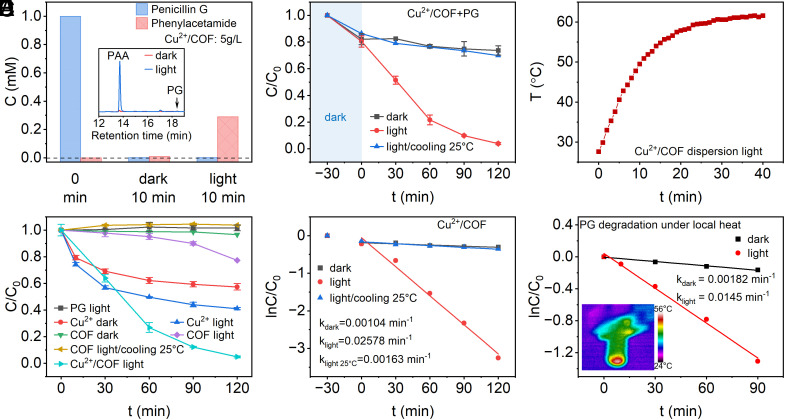
(*A*) The concentration of PG (C_0_ = 1 mM) and product PAA after being treated with a high concentration of Cu^2+^/COF (5 g/L) for 10 min under dark and light, the *Inset* was HPLC spectra of PG degradation. (*B*) PG (C_0_ = 0.1 mM) degradation performance of Cu^2+^/COF (0.2 g/L) under different conditions including dark, light, and light/cooling 25 °C. (*C*) A plot of the temperature of Cu^2+^/COF dispersion (0.2 g/L) within time under light. (*D*) PG degradation rate of COF (0.2 g/L), Cu^2+^ (0.045 mM, equal to Cu content of Cu^2+^/COF) and no catalyst (only PG) under different conditions including dark, light, and light/cooling 25 °C. PG degradation kinetics of *E* Cu^2+^/COF in dispersion and (*F*) Cu^2+^/COF under local photothermal conditions without stir, the *Inset* was an infrared thermal image of the reaction system. All of the light power above is 1 W/cm^2^.

To investigate the hydrolysis of β-lactam antibiotics by as-prepared Cu^2+^/COF, we choose Penicillin G (PG) as the first object to study in detail. When we used high dosage Cu^2+^/COF dispersion to hydrolyze very high-concentration PG water solution (1 mM), PG was almost completely hydrolyzed in 10 min regardless of irradiation or dark conditions (Fig. 3*A*) as PG peak disappeared in the High-performance liquid chromatography (HPLC) spectra (*Inset* in Fig. 3 *A*). However, the key hydrolyzed product, phenylacetamide (PAA), did not distinctly appear in the dark case (less than 1% yield), whereas a much higher concentration of PAA (0.293 mM, 29.3% yield) was observed for the irradiation case. This indicated Cu^2+^/COF had strong adsorption to PG and the intermediates even under the dark case, but only under irradiation could the adsorbed PG be faster hydrolyzed into PAA by Cu^2+^/COF. For more thorough studies, we set the initial PG concentration as 0.1 mM, which was closer to the pharmaceutical wastewater (55, 56). In such a situation, two problems must be addressed in advance. First, it needs to avoid the interference of the adsorption of PG as well as its hydrolyzed intermediates by Cu^2+^/COF to accurately track the real situation of PG hydrolysis. To this end, excess EDTA was added to the samples before HPLC analysis which can ensure to displace out targets completely from Cu^2+^/COF (detailed in *SI Appendix*, Fig. S8-1) (57). Second, by screening different Cu^2+^ load to COF and dosage of Cu^2+^/COF (*SI Appendix*, Fig. S8-2), the optimal theoretical load of Cu^2+^ is 4 wt% and the optimal dosage in solution is 0.2 g/L. Thus, the catalytic hydrolysis of PG with Cu^2+^/COF under different conditions was carried out (Fig. 3*B*) including dark, light irradiation (noted as light) and light irradiation but with a cooling control temperature of 25 °C (noted as light/cooling 25 °C). To make sure adsorption equilibrium between Cu^2+^/COF and PG, the dispersion was stirred for 30 min in the dark at room temperature. As expected, PG was degraded faster under illumination than that in dark case, the total removal efficiency was 96% over 120 min, with concomitant an overall temperature rising from an initial 27 °C to 62 °C. The temperature Versus time under light irradiation without any heat preservation measures is shown in Fig. 3*C*. Because there were identical degradation performances between the dark and light/cooling 25 °C and no other degradation pathways of PG else except hydrolysis (details see later section), the promoted removal performance under irradiation would be ascribed to thermal energy converted from incident photons. The control experiments including COF alone, homogenous Cu^2+^ (0.045 mM, equivalent to Cu^2+^/COF), and without any catalyst (only PG) under dark and light were carried out (Fig. 3*D*). The results furthermore excluded COF’s adsorption or photocatalysis function and the substrate itself photolysis. More significantly, our catalyst even presented better activity than Cu^2+^ homogeneous catalysis under illumination, which evidently broke through the convention that supports generally limited catalytic activity owing to anchoring having to occupy the catalytic sites. This implied the enhanced photothermal effect from support COF that elevated loaded Cu^2+^ activity. The plot of degradation kinetics following the pseudo-first-order model was fitted in Fig. 3*E*. The rate constant under irradiation is 0.0258 min^−1^, about 24-fold higher than that under dark (0.00104 min^−1^), very consistent with the relationship of the hydrolytic rate constant to temperature (24). The antibiotic pharmaceutical wastewater usually has a high chroma, we also conducted PG degradation tests under different chroma conditions adjusted by methyl orange dye. It was found that the photothermal conversion effect of Cu^2+^/COF material was little affected by the high chroma and the hydrolysis performance of PG could still mostly be maintained with the high chroma (*SI Appendix*, Fig. S8-3).

As mentioned earlier, the stirring accelerated the heat exchange between adsorbed water layers around Cu^2+^/COF and bulk solution and caused the catalytic sites to cool, which was absolutely unfavorable to the hydrolysis of PG. Therefore, we conducted PG hydrolysis under a local photothermal condition (reaction equipment same as Fig. 2*E*) instead of stirring in which Cu^2+^/COF powder was deposited on the bottom and exposed to light. The thermal image showed the high temperature was efficiently preserved in the Cu^2+^/COF body (*Inset* of Fig. 3 *F*). Limited mass transfer of PG without stirring certainly reduced the degradation performance relative to Cu^2+^/COF dispersion and the hydrolysis rate was only over half as much as the stirring case (0.0145 min^−1^ Versus 0.0258 min^−1^), but the local photothermal effect was confirmed to be still valid in enhancing the hydrolysis of PG by 10 times higher than that under dark (Fig. 3*F*) for complete hydrolysis of PG. When the volume of the reaction solution and the loading dosage of Cu^2+^/COF both were magnified by 15 times and even by 50 times (*SI Appendix*, Fig. S8-4), the degradation rate of PG under local photothermal conditions was still around 10 times than under darkness and the obvious local photo-to-heat effect remained. Except for PG, Cu^2+^/COF displayed photo-to-heat promoted degradation performance to other common β-lactam antibiotics, for instance, ampicillin (AMP), amoxicillin (AMX), cefalexin (CFX), and meropenem (MPN) (*SI Appendix*, Fig. S8-5). Their structures are shown in Fig. 1*A* (58). Their hydrolysis rate constants under light generally can improve about 10 to 20 times relative to dark cases (Table 1). The results unambiguously demonstrated that as-prepared Cu^2+^/COF could serve as an efficient photothermal-driven Lewis catalyst to hydrolyze different β-lactam antibiotics in water.

**Table 1.** Catalytic hydrolysis rate constants (min^−1^) of PG and other β-lactam antibiotics (C_0_ = 0.1 mM) by Cu^2+^/COF (0.2 g/L) under dark and light (1 W/cm^2^)

	Penicillin G	Ampicillin	Amoxicillin	Cefalexin	Meropenem
dark	0.00104	0.0014	0.0025	0.0019	0.0041
light	0.0258	0.0133	0.0217	0.0225	0.0378

### Products Identification.

**Fig. 4.**
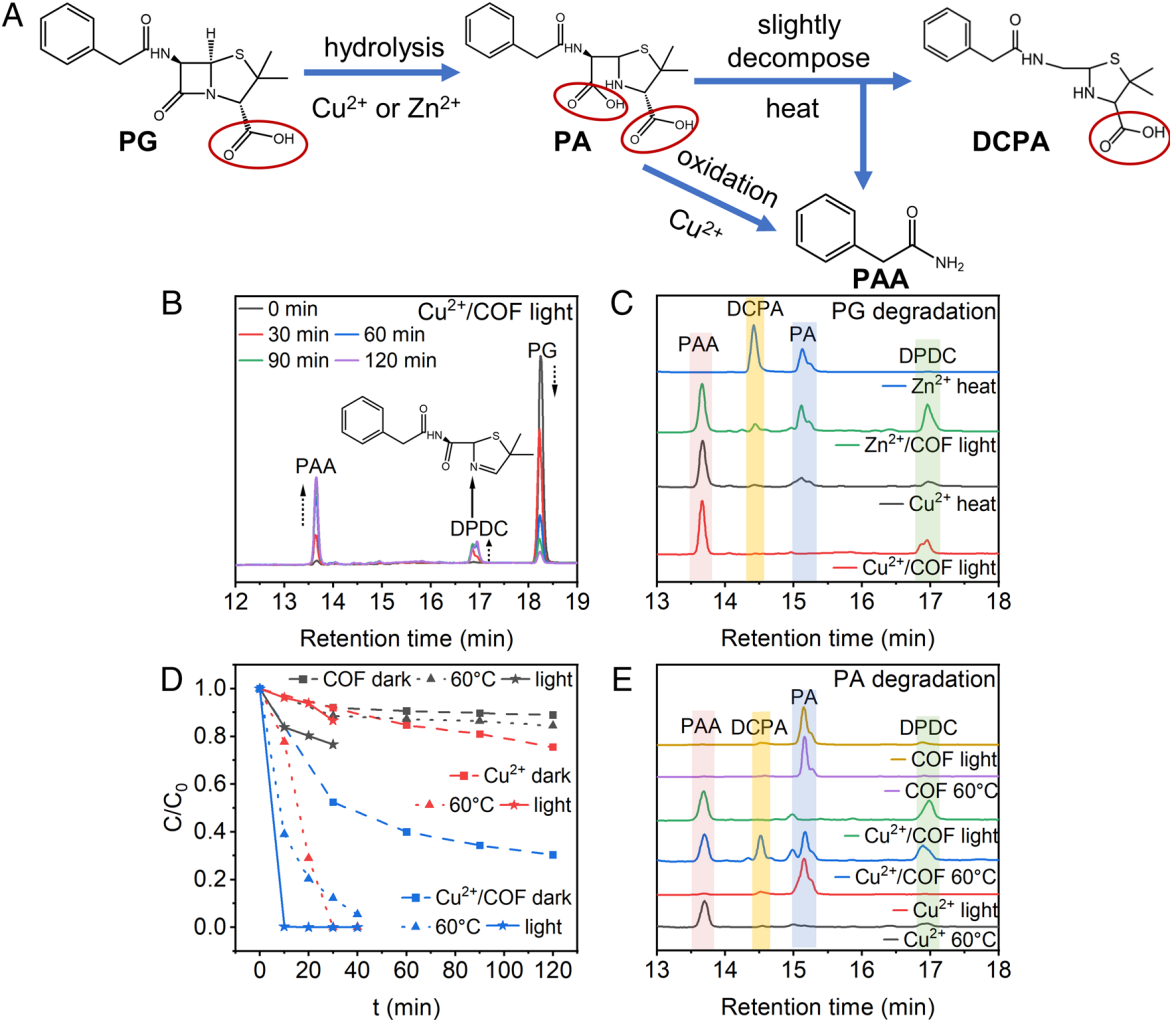
(*A*) The common homogeneous hydrolytic pathway of PG. (*B*) The PG degradation products LC spectra of Cu^2+^/COF photothermal catalysis at 30 min intervals. (*C*) LC spectra showed the different degradation products of PG-mediated between Zn^2+^, Cu^2+^ homogeneous catalysis, and Zn^2+^/COF, Cu^2+^/COF photothermal catalysis. (*D*) The PA degradation performance mediated by Cu^2+^ and Cu^2+^/COF under dark and light conditions and resulted in (*E*) different degradation intermediates determined by LC spectrum.

For general Lewis acid catalysis, the initial hydrolytic product of PG should be penicilloic acid (PA) (Fig. 4*A*). PA is relatively stable and usually does not undergo further transformation at room temperature. Only a small amount of decomposition to decarboxylated PA (DCPA) and PAA occurs even when heated (*SI Appendix*, Fig. S9-1). Different from other metal ions such as Zn^2+^, homogenous Cu^2+^ catalysis has been revealed to have the ability to partially convert PA to PAA because it has an oxidizing ability and easily runs O_2_-driven Cu^2+^/Cu^+^ redox (31, 59). Nevertheless, the complete decarboxylation for all hydrolyzed products through other oxidative pathways cleaving the –C-COOH bond has not yet been developed in detail. Here, we used HPLC-MS to systemically determine these possible intermediates and end-products in different catalytic systems and reaction time to reveal the extraordinary features of decarboxylation followed by or paralleled to the main hydrolysis by our Cu^2+^/COF photothermally catalyst (*SI Appendix*, Fig. S9-2 *A*–*D*). Indeed, whenever Cu^2+^/COF degraded PG, PAA as the main product was always detected, while no obvious PA and DCPA intermediates were observed (Fig. 4*B*). Such a result indicated that in analogous to other Cu^2+^-based catalysts, Cu^2+^/COF with photothermal effect possibly accelerated PG hydrolysis and further transformation of PA. In addition, beyond our expectation, a previously unreported decarboxylated product 5,5-dimethyl-N-(2-phenylacetyl)-2,5-dihydrothiazole-2-carboxamide (noted as DPDC, MW 276, its characteristic seen in *SI Appendix*, Fig. S9-2 *E* and *F*) was generated throughout the reaction period. We argued that DPDC likely stemmed from the simplest hydrolysis product PA which may disappear too fast to be observed in our present Cu^2+^/COF system. Therefore, we contrasted the reaction products of PG in Zn^2+^, Cu^2+^ homogeneous catalysis and Zn^2+^/COF heterogeneous systems under otherwise identical conditions (Fig. 4*C*). As expected, Zn^2+^ ion as a normal Lewis acid catalyst without redox ability (difficult to run O_2_-driven Zn^2+^/Zn^+^ cycle) catalyzed PG hydrolysis just to generate PA and DCPA, but never generated PAA. On the contrary, when free Cu^2+^ ion as a homogeneous catalyst was used for the hydrolysis of PG, the main products were PAA and DPDC accompanied by less PA as well as DCPA. This indicated that the catalytic conversion of PA by Cu^2+^ is incomplete and slower than that of Cu^2+^/COF, leading to both PA and DCPA coexistence in the solution. Unexpectedly, PAA and DPDC were produced in the Zn^2+^/COF photothermal degradation process. These results indicate that the coordination of metal ions and COF has a synergistic effect on the PA complete decarboxylation transformation. To confirm this proposal, we furthermore took PA as the initial substrate to perform the degradation experiments with Cu^2+^, COF, and Cu^2+^/COF under dark, light and heat, respectively (Fig. 4*D*). The PA degradation rate increased drastically when Cu^2+^ was combined with COF under no matter what condition and the products were also PAA and DPDC (Fig. 4*E*). As well, we compared the ability of PA transformation with Zn^2+^ and Zn^2+^/COF under light and heat conditions (*SI Appendix*, Fig. S9-3*A*). It can be found that Zn^2+^/COF can transform PA, and the end products (PAA and DPDC) were completely decarboxylated. On the contrary, Zn^2+^ alone can’t do any. This means that there must be another way of full-decarboxylation of hydrolyzed intermediate mediated by this Cu^2+^/COF photothermal excitation, not just the traditional O_2_-driven Cu^2+^/Cu^+^ route (*SI Appendix*, Fig. S9-5*A*).

**Fig. 5.**
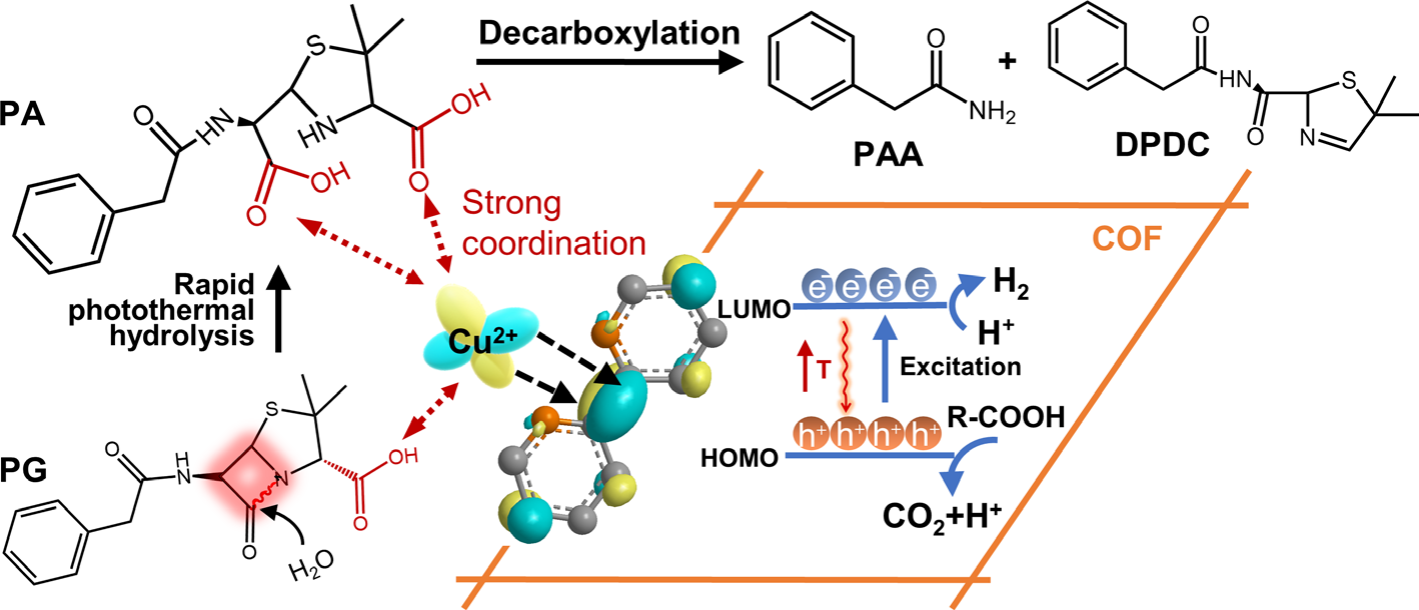
The full-decarboxylation of intermediates paralleled to hydrolysis of PG mediated by the photothermal Cu^2+^/COF catalyst.

To explore why our Cu^2+^/COF combination enables primary hydrolyzed product PA full decarboxylation accompanied with main hydrolysis reaction, we compared the FTIR of PG and PA interacted with COF, Cu^2+^, Zn^2+^, Cu^2+^/COF, and Zn^2+^/COF, respectively, and found that the interaction between M^2+^/COF and carboxyl groups of both PG parent and PA intermediate was significantly enhanced compared with homogeneous metal ions (*SI Appendix*, Fig. S9-4 and *Text S11*). This indicated that Cu^2+^/COF and Zn^2+^/COF are precisely conducive to decarboxylation after the main hydrolysis of the β-lactam bond. Due to the existence of a prominent feedback π bond from Cu^2+^ to 2, 2′-bipyridine molecule (60), HOMO and LUMO orbit in M^2+^/COF are more prone to charge separation under light or heat excitation. Such a charge separation state endows the holes and electrons formed on HOMO and LUMO to execute redox decarboxylation in parallel to the photothermally driven main hydrolysis reaction, which is likely through stabilizing the carbanion intermediates by enhanced positive charge M^2+^ component (Fig. 5). We further compared the degradation product changes of Zn^2+^/COF under both light irradiation and heating conditions. It was found that for the Zn^2+^/COF case, both PAA and DPDC were enhanced under photothermal conditions, while sole PAA generation from PA was dominated by heating. (*SI Appendix*, Fig. S9-3 *B* and *C*). Accordingly, a more detailed hydrolysis/decarboxylation parallel approach along the photothermal-driven charge separation on M^2+^/COF construction could be drawn in *SI Appendix*, Fig. S9-5*B* and Text S12.

### The Capacity of Antidisturbance and Recyclability.

**Fig. 6.**
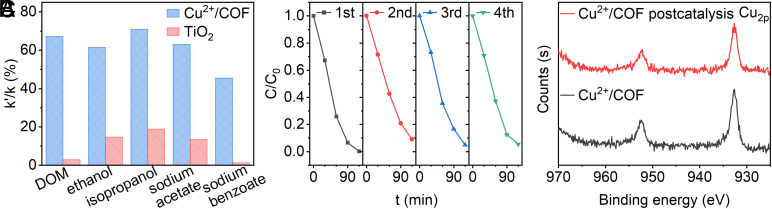
(*A*) The ratio of rate constant with interferent (noted as k′) to without interferent (noted as k) between the photocatalytic degradation method with P25-TiO_2_ (0.2 g/L, 100 mW/cm^2^ UV light/cooling 25 °C) and photothermal degradation method with Cu^2+^/COF (0.2 g/L, 1 W/cm^2^ sunlight), in which interferent was DOM (0.05 mM), ethanol, isopropanol, sodium acetate, and sodium benzoate (100 mM), respectively. (*B*) The recyclability of Cu^2+^/COF under the photothermal condition, the concentration of catalyst (0.2 g/L), and the reaction volume (20 mL) were the same in each run. (*C*) The Cu_2p_ XPS spectra of Cu^2+^/COF before and after photothermal degradation.

In general, industrial wastewater contains a large amount of DOM and other artificial organics which will significantly compete with the target contaminants degradation, especially for these ROS-based catalytic technologies. So we separately choose water-soluble humic acid as DOM and ethanol, isopropanol, acetate, and benzoate salt as artificially effluent pollutants to compare their impact on our system with the representative TiO_2_ photocatalysis based on ROS. The concentration of DOM and the other interferents was controlled at 0.05 mM and 100 mM, respectively, which was close to the actual wastewater features. *SI Appendix*, Fig. S10 showed the removal efficiency versus irradiation time when adding diverse interferents. The ratio of rate constant with (noted as k′) and without interferent (noted as k) was used to judge influenced degree. Fig. 6*A* contrasted these values between P25-TiO_2_, UV photocatalytic degradation and Cu^2+^/COF photothermal degradation. As expected, the degradation of PG by P25 photocatalyst was drastically inhibited since major ROS such as •OH radical were intercepted by interferences, whereas our photothermal catalysis method performed a much better capacity of antidisturbance. As for different coexisting organics, Cu^2+^/COF made PG hydrolysis robust and more resistant to interferences, most holding 60 to 70% efficiency relative to without interference. The loss of hydrolysis efficiency, for instance of the benzoate case, we ascribed to its more or less competing complex with the Cu^2+^ moiety of Cu^2+^/COF that suppressed PG interaction with Cu^2+^/COF. To further demonstrate the potential superiority in practical application, we compared the degradation performance of Cu^2+^/COF photothermal hydrolysis with other common AOPs including TiO_2_-based photocatalysis, Fenton and O_3_ oxidation treatments in the presence of glucose, butyl acetate, phenylacetic acid interferences (*SI Appendix*, Fig. S11), which were the main components of penicillin fermentation production wastewater. The results again proved that the photothermal hydrolysis method did have better antiinterference ability than the conventional AOPs pretreatment technologies. Although far outweighed the common AOT treatments, the antiinterference ability of Cu^2+^/COF assembly needs to be further improved by designing better COF construction for Cu^2+^ ion coordination and Lewis acid catalysis.

To evaluate the stability of Cu^2+^/COF during long-time photothermal hydrolysis, we used ICP-MS to determine the amount of Cu^2+^ leached from Cu^2+^/COF into the solution. After one photothermal run, only ~2% of the total Cu load was found in the solution, which was suspected to come from the physical adsorption of Cu ions in the internal pore of COF. The recyclability of Cu^2+^/COF photothermal catalytic PG degradation was examined (Fig. 6*B*). The performance of the fourth run remained with the first run, which actually proved the stability of the catalyst. Simultaneously, we used XPS to examine the binding energy of Cu_2p_ (Fig. 6*C*), it was found that after the reaction, the binding energy of Cu_2p_ was the same as the original Cu^2+^/COF, demonstrating the chemical state of Cu^2+^ was certainly unchanged before and after use.

In summary, we provided a unique β-lactam antibiotics degradation method by using the photothermal conversion effect of Cu^2+^/COF to significantly promote hydrolysis of the most fatal β-lactam rings. In the successful assembly, Cu^2+^ ion was solidly coordinated into support through the Bpy unit of Py-Bpy-COF, which not only still holds its strong Lewis acidity but also remarkably broadens Py-Bpy-COF absorption range toward the visible region thereby enhancing photothermal hydrolysis of β-lactam rings. Product identification indicated that degradation was through Lewis acid-catalyzed hydrolysis-decarboxylation parallel approach strengthened by the extraordinary charge separation on Cu^2+^/COF structure. Such a photothermal hydrolysis strategy certainly owned an exceptional capacity of antidisturbance compared with the other photocatalytic methods. This photothermally catalyst for the specific degradation of β-lactam antibiotics can be feasibly extended to the degradation of other contaminants that are sensitive to temperature.

## Materials and Methods

### Chemicals and Materials.

The chemicals used in this study were described in *SI Appendix*, *Text S1*. Py-Bpy-COF and Cu^2+^/COF of different content [noted as Cu^2+^/COF(x wt%)] were synthesized following the method of Guo (46) and Zhang (40), as shown in *SI Appendix*, Fig. S1. The details were also shown in *SI Appendix*, *Text S2*.

### Characterization.

The detailed characterization description is shown in *SI Appendix*, *Text S3*.

### Photothermal Conversion Performance.

The temperature and thermal imaging were detected on a FLUKE TIX580 camera. The light source was a Xe lamp with an AM1.5 optical filter (PLS-SXE300D) and the optical power density was 1 W/cm^2^. The photothermal conversion efficiency (η) was calculated based on the following formulas (see *SI Appendix*, *Text S4* for details) (61).

$$\eta =\frac{hA{\Delta T}_{max}-Q_{s}}{q}.$$

### Degradation Test of β-Lactam Antibiotics.

A certain amount of powder sample was dispersed in β-lactam antibiotics solution (0.1 mM) and stirred for some time in the dark. Then, the experiments were conducted under different conditions including dark, light irradiation, and light irradiation but with a cooling control temperature of 25 °C. The light source was simulated sunlight (generated by a 300 W Xe lamp with an AM1.5 filter) and its optical power density was 1 W/cm^2^. At each time interval, 1 mL of suspension was sampled and tested after direct filtration or EDTA extraction. In the EDTA extraction experiment, 0.1 mL of ethylenediaminetetraacetic acid dipotassium salt (EDTA) solution (20 mM) was spiked into samples. The mixture was shaken for 2 min to extract the adsorbed antibiotics and filtered through a 0.22-μm poly(tetrafluoroethylene) membrane. In addition, the post-treated samples were stored in 2-mL amber vials at 5 °C and analyzed within 24 h. In the recycling experiment (Details in *SI Appendix*, *Text S13*), Cu^2+^/COF was collected by filtration. After washing once with H_2_O, the catalyst was put in the next run. A pseudo-first-order kinetics model was used to fit the degradation kinetics, and then, the rate constant (k, min^−1^) was calculated. Calculation details of the pseudo-first-order degradation constant were involved in *SI Appendix*, *Text S5*.

During P25 photocatalytic degradation process, the concentration of P25 and PG was 0.2 g/L and 0.1 mM. The light source was UV light (200 to 400 nm, generated by a 300 W Xe lamp with a UVREF filter) and its optical power density was 100 mW/cm^2^. The reaction was conducted at 25 °C, and samples were tested after direct filtration. The Fenton process was conducted at pH 4 and the concentration of Fe^2+^ and H_2_O_2_ was 0.045 mM and 9 mM, respectively. Ozone was generated from O_2_ by an ozone generator (UVP SOG-2). Ozonation was performed by continuously bubbling the ozone/O_2_ mixture into the solution at a flow rate of 30 mL/min and the ozone concentration was 30 ppm.

### Analytical Methods of Antibiotics.

Details of the analytical methods of antibiotics are described in *SI Appendix*, Table S1 and *Text S6*.

## Supplementary Material

Appendix 01 (PDF)

## Data Availability

All study data are included in the article and/or *SI Appendix*.
