# Supplementary material for: Cu2+ coordination-induced in situ photo-to-heat on catalytic sites to hydrolyze β-lactam antibiotics pollutants in waters
Source: Proc Natl Acad Sci U S A. 2023 Dec 18;120(52):e2302761120. doi: 10.1073/pnas.2302761120 (PMC10756305; doi:10.1073/pnas.2302761120)
Supplement: Supplementary file 1 — Appendix 01 (PDF) [file pnas.2302761120.sapp.pdf]

PNAS

Supporting Information for
Cu^{2+} coordination-induced in situ photo-to-heat on catalytic sites to hydrolyze β-lactam antibiotics pollutants in waters

Jiazhen Li[a,b], Dongge Ma[c]*, Qiang Huang[a,b], Yangyang Du[a,b], Qin He[a,b], Hongwei Ji[a,b]*, Wanhong Ma[a,b]*, Jincai Zhao[a,b]

[a]Key Laboratory of Photochemistry, Beijing National Laboratory for Molecular Sciences, Institute of Chemistry, Chinese Academy of Sciences, Beijing 100190, People's Republic of China
[b]School of Chemical Sciences, University of Chinese Academy of Sciences, Beijing 100049, People's Republic of China
[c]Department of Chemistry, College of Chemistry and Materials Engineering, Beijing Technology and Business University, Beijing 100048, People's Republic of China

*Wanhong Ma, Hongwei Ji, Dongge Ma
Email: whma@iccas.ac.cn, hwji@iccas.ac.cn, madongge@btbu.edu.cn

This PDF file includes:

Supporting text
Figures S1 to S11
Tables S1 to S3
SI References

Supporting Information Text

Text S1. Chemicals

1,3,6,8-Tetrakis(4-aminophenyl)pyrene and 5,5'-Diformyl-2,2'-bipyridine were purchased from Shanghai Chemsoon. Copper(II) acetate monohydrate, Sodium acetate, Sodium benzoate, mesitylene, dioxane, Benzylpenicillin potassium, Ampicillin, Amoxicillin, and Meropenem were obtained from Inno-Chem. Acetate, ethanol, methanol, isopropanol and N, N-Dimethylformamide were purchased from Concord Technology. Cefalexin was gotten from J&K. All the chemicals are analytical grade and used without further purification. Deionized water was prepared with a Milli-Q purification system and used throughout all the experiments.

Text S2. Synthesis of Cu^{2+}/Py-Bpy-COF

A mesitylene/dioxane/6 M AcOH (5/5/1 by vol., 3.3 mL) mixture of 1,3,6,8-Tetrakis(4-aminophenyl)pyrene (Py, 67.8 mg) and 5,5'-Diformyl-2,2'-bipyridine (Bpy, 51 mg) in a solvent storage bottle (Schlenk, 10 mL) was degassed by three freeze-pump-thaw cycles and refilled with Ar. The bottle was heated at 120 °C for 7 days. The precipitate was collected by filtration and washed with DMF 3 times and ethanol 3 times. The powder was dried at 60 °C under a vacuum oven overnight to give the Py-Bpy-COF (noted as COF). 100 mg of the as-prepared COF was dispersed in 10 mL methanol and calculated amount of $Cu(OAc)_2 \cdot H_2O$ (3 mg (1 wt%), 6 mg (2 wt%), 12 mg (4 wt%), 18 mg (6 wt%)) dissolved in 10mL methanol. Then $Cu(OAc)_2$ solution was added dropwise to COF dispersion. The mixture was stirred for 4 h at room temperature, following which it was washed with water 3 times and methanol 3 times. The obtained material named Cu^{2+}/COF (x wt%) was dried using a vacuum oven overnight at 60 °C.

Text S3. Characterization

Scanning electron microscope (SEM) images were taken on Hitachi SU 8020. Transmission electron microscopy (TEM) images and energy dispersive X-ray (EDX) analysis elemental maps were taken on an FEI Tecnai G2 F20 U-TWIN microscope. X-ray photoelectron spectroscopy (XPS) was conducted on Thermo Fisher Scientific ESCALAB250XI X-ray photoelectron spectrometer with a non-monochromatized Al Kα X-ray source (1486.6 eV). Powder X-ray diffraction (PXRD) patterns of the samples were recorded on a Malvern Panalytical Empyrean X-ray powder diffractometer operated at 40 kV voltage and 40 mA current with CuKα radiation (λ = 1.5406 Å). UV–vis diffuse reflectance spectrum (DRS) was obtained in a UV–vis spectrophotometer (HITACHI UH-4150) using a barium sulfate tablet as the absorb standard. Fourier-transformed infrared spectra (FT-IR) were collected on a Bruker Vertex 70 V spectrometer equipped with a narrow band HgCdTe detector. Thermal images were taken on an infrared thermal-imaging camera thermographic system with an accuracy of 0.1 °C (Fluke, TIX580-9HZ/CN). Metal content was quantified by Inductively coupled plasma mass spectrometry (Thermo iCAP RQ). The N_2 adsorption/desorption isotherms were measured at 77 K by Quantachrome Autosorb iQ. And the samples were outgassed at 120 °C for 24 h before measurement. Surface areas were calculated based on Brunauer-Emmett-Teller (BET) method. Pore size distribution curves were obtained via the non-local density functional theory (NLDFT) method. Thermogravimetric analysis (TGA) was conducted on a TG-DTA6300 instrument under an N_2 atmosphere. ^{13}C and ^{1}H solid-state nuclear magnetic resonance (ssNMR) spectra were collected on a Bruker NEO 600 WB instrument. Steady fluorescence spectra were measured by HITACHI F7000. Fluorescence lifetimes were measured using an FLS1000 Edinburgh Instruments spectrofluorometer. X-ray absorption near-edge structure (XANES) and Extended X-ray absorption fine structure (EXAFS) data were collected at the Beijing Synchrotron Radiation Facility (BSRF), with the raw transmission mode data processed via background-subtraction, normalization, Fourier transformations and fitting using Athena and Artemis software (1). Fourier transform infrared (FT-IR) spectroscopy was performed by ATR mode on a Bruker Vertex 70 V spectrometer equipped with a narrow band HgCdTe detector.

The femtosecond transient absorption (TA) spectra were recorded on a commercial ultrafast Transient Absorption Spectrometer (HARPIA-TA) in combination with a femtosecond laser amplified system (PHAROS) and optical parametric amplifier (ORPEHUS-HP). Laser pulses (~1030 nm center wavelength, < 230 fs, 100 kHz repetition rate) were generated by a Ti: Sapphire-

based regenerative amplified laser system. The OPA was used as the pump source tuned to an output of 395 nm and yielding ~50 fs pulses. The probe and reference beams were generated by focusing the 1030 nm beam (split from the amplifier with a tiny portion) onto a sapphire crystal to generate white-light continuum pulses (490 - 900 nm).

The Mott-Schottky analysis was conducted on an electrochemical workstation (Autolab, PGSTAT 302 N, Metrohm) in a standard three-electrode one-compartment (20 mL) configuration with the photocatalyst-coated FTO as the working electrode (WE), Pt wire as the counter electrode (CE), and Ag/AgCl as the reference electrode (RE). Besides, 0.25 M of Na_2SO_4 solution was used as the electrolyte solution. Electrochemical test was then conducted at frequencies of 500, 750 and 1000 Hz, respectively. The WE was prepared according to the following procedure, 5 mg of the photocatalyst was added to a mixture of Nafion (10 μL) and ethanol (1 mL). Next, the obtained suspension (160 μL) was dropped on the surface of the FTO substrate with a working area of 2 × 2 cm^2.

Text S4. Photothermal conversion efficiency measurement

Based on the total energy balance for this system:

$$\sum_i m_i c_{p,i} \frac{dT}{dt} = Q_{cat} + Q_s - Q_{loss} \quad (1)$$

$$\eta = \frac{hA\Delta T_{max} - Q_S}{q} \quad (2)$$

where Q_{cat} is thermal energy raised from the catalyst under irradiation, Q_s is the heat associated with the light absorbance of the solvent, which is measured independently using pure water without catalyst and q is nominal of direct solar irradiation on the material.

Q_{loss} is thermal energy lost to the surroundings:

$$Q_{loss} = hs\Delta T \quad (3)$$

where h is the heat transfer coefficient, s is the surface area of the container, and ΔT is the temperature change, which is defined as $T\text{-}T_{surr}$ (T and T_{surr} are the solution temperature and ambient temperature of the surroundings, respectively).

At the maximum steady-state temperature, the heat input is equal to the heat output, that is:

$$Q_{cat} + Q_s = Q_{loss} = hs\Delta T_{max} \quad (4)$$

where ΔT_{max} is the temperature change at the maximum steady-state temperature. hs can be determined by applying the linear time data from the cooling period vs. –ln θ.

$$t = -\frac{\sum_i m_i c_{p,i}}{hs} \ln\theta \quad (5)$$

$$\theta = \frac{\Delta T}{\Delta T_{max}} \quad (6)$$

Text S5. Kinetics fitting

The degradation kinetics of β-lactam antibiotics was modeled by the pseudo-first-order kinetic equation (1).

$$\ln(C/C_0) = -k \times t \quad (1)$$

Where C_0 and C are the initial concentration (mM) and the concentration of antibiotics (mM) at the interval time, respectively. k is the degradation constant of antibiotics (min^{-1}). The value of k for penicillin degradation could be obtained by fitting the variation of C/C_0 vs. reaction time (min).

Text S6. Analytical Methods

HPLC method parameters for the detection of various β-lactam antibiotics are shown in Table S1. β-lactam antibiotics were analyzed by Spursil C18 column (4.6 × 250 mm, 5 μm) except for PG (Diamonsil C18(2) column (3.0 × 250 mm, 5 μm)). Transformation products of PG were analyzed by an Agilent 1100 HPLC system and Thermo Scientific Fusion Lumos LC-MS system with a Diamonsil C18(2) column (3.0 × 250 mm, 5 μm). Gradient elution was carried out using 0.1% phosphoric acid (formic acid, as for LC-MS) in water (A) and pure acetonitrile (B) at a flow rate of 0.3 mL/min: 10% B was kept for 2 min, then ramped to 50% B over 8 min, kept for 5 min, and finally ramped back to 10% B. The injection volume was 20 μL. The products were analyzed by electrospray ionization at positive mode (ESI+) with a mass scan range of m/z 50−1000. Other parameters were set as follows: drying gas 8 L/min at 350 °C and nebulizer pressure 40 psi.

Text S7. Structure and Morphology

Figure S2a showed FT-IR spectra of precursor Py, Bpy and COF, the band at 3425 cm^{-1}, 3369 cm^{-1}, 1616 cm^{-1} and 1697 cm^{-1} corresponding to the amine (-NH_2) of Py and aldehyde (-CHO) of Bpy respectively. The band at 1622 cm^{-1} was ascribed to the formed imine (C=N) (2). The change of IR signal demonstrated the successful synthesis of imine-linked Py-Bpy-COF. The PXRD of the as-prepared COF and different content Cu^{2+}/COF were shown in **Figure S2b**. PXRD results showed Py-Bpy-COF had good crystallinity with diffraction peaks at 3.2°, 4.6°, 6.4°, 9.7°, and 12.9°, which were attributed to the (110), (020), (220), (330) and (440) facets, respectively (2). The remained diffraction peaks after copper modification implied that the structure of Py-Bpy-COF wasn't affected by Cu^{2+}. ICP-MS analysis (**Table S2**) showed the actual copper load of different theoretical content Cu^{2+}/COF. **Figure S3-1 a-b** showed the SEM images of COF and Cu^{2+}/COF, the size of both COF and Cu^{2+}/COF was micrometer scale. Their TEM images (**Figure S3-1 c-f**) showed obvious lattice fringe and the lattice spacings both were 1.9 nm which was consistent with the (110) facet. **Figure S3-1g** showed the HAADF−STEM image and the corresponding EDS elemental mapping of Cu^{2+}/COF to verify the successful load of Cu^{2+}. X-ray photoelectron spectroscopy (**Figure S3-2**) was performed to further determine the coordination site of Cu^{2+}. The deconvoluted N 1s spectra of COF indicated the presence of two distinct peaks: 399.7 eV, corresponding to the pyridinic nitrogen (3), and 399.1 eV, which is associated with the imine nitrogen (4). The deconvoluted N 1s spectra of Cu^{2+}/COF showed an extra blue-shifted peak at 400.25 eV implying Cu^{2+} was docked with partial bipyridine. The solid NMR showed in **Figure S3-3**, ^{13}C chemical shift had no change between COF and Cu^{2+}/COF again verifying the structure remained. And a new peak at 1.16 ppm appeared in ^{1}H NMR of Cu^{2+}/COF ascribing to the chemical shift of the H atom in $Cu(OAc)_2$.

X-ray absorption spectroscopy (XAS) measurements were conducted to figure out the electronic structure of Cu ion in Cu^{2+}/COF. The raw transmission mode data was processed via background subtraction, normalization, Fourier transformations and fitting by using Athena and Artemis software. The Cu K-edge X-ray absorption near-edge structure (XANES, **Figure S3-4a**) of Cu^{2+}/COF was close to CuO and copper phthalocyanine (CuPc), indicating the valence state of Cu was approximately +2. As shown by the extended X-ray absorption fine structure (EXAFS, **Figure S3-4b**), the typical peak of the Cu–Cu metal bond (Cu foil) was absent in Cu^{2+}/COF and the peak (~1.94 Å) of Cu^{2+}/COF at the first shell was close to Cu-O/N in CuO and CuPc. Verified by EXAFS fitting curve (**Figure S3-4 c-j**) and parameters (**Table S3**), the Cu^{2+} sites in our Cu^{2+}/COF were atomically dispersed and coordinated with four N/O atoms. Although XAS was difficult to distinguish between Cu-N and Cu-O, Cu^{2+} should coordinate with two N atoms and two O atoms according to the composition of COF and copper salt.

Nitrogen sorption isotherms of COF and Cu^{2+}/COF were measured at 77 K (**Figure S3-5**), the Brunauer–Emmett–Teller (BET) surface areas were calculated to be 2061 m^2/g and 1985 m^2/g for COF and Cu^{2+}/COF. Their total pore volumes were 1.56 cm^3/g and 1.29 cm^3/g respectively. The slightly decreased specific surface area and pore volume of Cu^{2+}/COF were due to Cu^{2+} occupying on COF surface. We evaluated the pore size distributions by using the nonlocal density function theory (NLDFT) method, they both had a main peak at around 1.8 nm. Thermogravimetric analysis (**Figure S3-6**) suggested that COF and Cu^{2+}/COF had good thermal stability.

Text S8. The Band-structure characterization

The optical band gap can be calculated from the equation $(\alpha h\nu)^{1/n} = A(h\nu - E_g)$, where α stands for absorption coefficient proportional to absorbance so the Tauc plots were obtained by replacing α with absorbance. The hv value at the intersection point between the tangent line in linearity region and the horizontal axis is the band gap (E_g). As **Figure S4a** showed, the E_g of COF and Cu^{2+}/COF was 2.26 eV and 1.80 eV. The Mott-Schottky plots (**Figure S4 b-c**) were collected at the frequencies of 500, 750 and 1000 Hz. The positive values of the slope of C^{-2} vs. Potential demonstrated COF and Cu^{2+}/COF were n-type semiconductors. And the intersection point of the three plots was Fermi level. The Fermi level is -0.7 V and -0.83 V (vs. Ag/AgCl) for COF and Cu^{2+}/COF. The energy difference between the Fermi level and the valence band maximum (VBM)

of COF (2.20 eV) and Cu^{2+}/COF (1.67 eV) can be obtained by linear extrapolation of the leading edge of the VB XPS spectrum to the extended baseline (**Figure S4d**). Based on the formula (E (vs. NHE) = E (vs. Ag/AgCl) + 0.1976), the energy level of VBM and conduction band minimum (CBM) was deduced (**Figure S4e**).

Text S9. The photophysical property of Cu^{2+}/COF and COF

COF consisted of Py and Bpy monomers and the fluorescence of COF (at 555 nm) was red-shifted and enhanced compared with the monomer Py (490 nm) due to intramolecular charge transfer (ICT) from Py to Bpy in the excited state (**Figure S5-1a**). The fluorescence of COF bulk was significantly quenched and blue-shifted after loading Cu^{2+}, causing dual emissions at 518 nm and 550 nm, respectively. The quenching of fluorescence indicated that the absorbed incident light was converted into heat, but this was not obviously caused by the single excited state relaxation. We speculated that fluorescence from the locally excited (LE) state and ICT state coexisted in Cu^{2+}/COF due to the relaxation from the LE state to the ICT state inhibited partially by Cu^{2+} (detail drawing Jablonski diagram in **Figure S5-2**). It was supported by the difference in their fluorescence lifetimes (**Figure S5-1 b-d**), in which Cu^{2+}/COF (τ ~0.27 ns) was shorter than Py (τ ~1.6 ns) but longer than COF (τ~ 0.19 ns). To in-depth illustrate their photothermal conversion effect, femtosecond transient absorption (TA) spectra of COF (**Figure S5-1e**) and Cu^{2+}/COF (**Figure S5-1f**) were conducted to investigate the ultrafast nonradiative transition. There was an obvious excited state absorption region around 600~700 nm, which was attributed to the ICT state absorption of COF and Cu^{2+}/COF, respectively. The kinetic decay curves and fitting results at maximum absorption wavelength for COF (675 nm) and Cu^{2+}/COF (700 nm) were shown in **Figure S5-1g**. As for COF, the ICT state had three non-radiation deactivate pathways including vibration relaxation (τ_1), internal conversion (IC) to the ground state (τ_2) and intersystem crossing (ISC) to the spin-triplet ICT state (τ_3). Cu^{2+}/COF also went through these three processes, but their lifetimes were all significantly shorter than that of COF, clearly indicating Cu^{2+}/COF was more beneficial to photothermal conversion.

Text S10. The local photo-to-heat PG degradation test.

Firstly, 1 mg Cu^{2+}/COF was dispersed in water, then Cu^{2+}/COF was deposited on a 1 cm hydrophilic filter membrane (FM) by suction filtration. The FM was transferred to the bottom of vessel with Cu^{2+}/COF side adown. Compress the FM to make Cu^{2+}/COF cling to the bottom. Next, 0.1 mM PG solution (4 mL) was poured slowly into the vessel and the local photo-to-heat degradation test proceeded under illumination from the bottom. 0.1 mL solution was taken at each time interval and analyzed by liquid Chromatogram. As for the scale-up experiment of magnified to 15 times and 50 times, a suspension containing 15 mg and 50 mg Cu^{2+}/COF was deposited on 5.5 cm and 9.5 cm FM, respectively. And we utilized double-faced adhesive tape to guarantee FM fixed on the bottom of vessel. Simultaneously, the PG solution was extended to 60 mL and 240 mL.

Text S11. FTIR analysis.

D_2O was used as a solvent of PG solution to avoid the interference of the strong H−O−H bending absorption on the IR absorption of aimed -COO-Cu^{2+}, -C=O-Cu^{2+}coordination, etc. The absorption bands of PG solution at 1762 cm^{-1}, 1637 cm^{-1}, 1602 cm^{-1} and 1402 cm^{-1} correspond respectively to the stretching vibration of ketone in the β-lactam ring ($v_{C=O}$), stretching vibration of ketone in amide bond ($v_{C=O,\ amide}$), asymmetric stretching vibration of carboxyl (v_{COOas}) and symmetric stretching vibration of carboxyl (v_{COOs}). The peak at around 1450 cm^1 was not analyzed due to the influence of D_2O. In addition, it is well known that the difference between the asymmetric and symmetric stretching vibrations of carboxyl groups Δv (Δv = $v_{COOas}-v_{COOs}$) can be used to identify carboxyl groups bind to metal ions with monodentate (Δv increase) or bidentate (Δv decrease) model.

Firstly, adding COF support without loading Cu^{2+} or Zn^{2+} to PG substrate solution for 15 min equilibrium showed no significant changes of main peaks of PG molecule except a little shift of peak ($v_{C=O,\ amide}$, from 1637 to 1641 cm^{-1}) (see **Figure S9-4a**), indicating no significant interaction with these function groups of PG, especially carboxyl groups. After the addition of homogeneous

Cu^{2+} ions to the PG substrate solution, there are no significant changes except a shift of peak ($v_{C=O,\ amide}$) from 1637 to 1631 cm^{-1} (see **Figure S9-4b** bottom), indicating that Cu^{2+} mainly coordinates with the carbonyl group ($v_{C=O,\ amide}$) in PG. Especially, there is no Δv ($v_{COOas} - v_{COOs}$ = 1603 cm^{-1} - 1403 cm^{-1} = 200 cm^{-1}) change relative to that of pure PG substrate case (Δv = 1602 cm^{-1} - 1402 cm^{-1} = 200 cm^{-1}), indicating no any significant interaction between Cu^{2+} ion and the carboxyl group of PG. We highly wonder if this weak interaction manifestation renders classic Lewis hydrolysis catalysts such as Zn^{2+} and Cu^{2+} unable to fully decarboxylation both the hydrolyzed intermediate and initial carboxyl at thiazolidine. Given this, Zn^{2+}, which acts as a more onefold Lewis hydrolysis catalyst without any redox property, is used to perform the identical ATR-FTIR measurement of PG. Indeed, it did show a highly consistent Δv shift ($v_{COOas} - v_{COOs}$ = 1603 cm^{-1} - 1403 cm^{-1} = 200 cm^{-1}) with the case of Cu^{2+} case relative to that of pure PG substrate case (see **Figure S9-4c** bottom). The striking difference is that when Cu^{2+}/COF is introduced into the identical ATR-FTIR measurement, there is a markedly enhanced interaction between Cu^{2+}/COF and carboxyl group in PG substrate beside the more shift of peak ($v_{C=O,\ amide}$) from 1637 cm^{-1} to 1653 cm^{-1} than that of Cu^{2+} case (see **Figure S9-4b** top) and, the increase of Δv from 200 cm^{-1} of pure PG case to 209 cm^{-1} indicates monodentate coordination between them has formed. We believe that both enhanced interactions are in line with the acceleration of the main hydrolysis and full decarboxylation performance of Cu^{2+}/COF. Similarly, for Zn^{2+}/COF, the interaction with carboxyl groups (Δv = 1606 cm^{-1} - 1403 cm^{-1} = 203 cm^{-1}) and ketone in amide bond ($v_{C=O,\ amide}$) is also increased compared with that of pure PG substrate case (see **Figure S9-4c** top). It should be attributed to the feedback π between Cu^{2+} (or Zn^{2+}) and N, N'-bipyridine of COF that enhances the interaction between Cu^{2+} or Zn^{2+} and carboxyl groups in PG, which is more conducive to the decarboxylation reaction. We further compared the ATR-FTIR spectra of primary intermediate PA combined with COF, Cu^{2+}, Zn^{2+}, Cu^{2+}/COF and Zn^{2+}/COF, respectively (see **Figure S9-4d, e** and **f**). The absorption bands of PA solution at 1597 cm^{-1} and 1385 cm^{-1} correspond to the asymmetric and symmetric stretching vibration vibrations of carboxyl groups (Δv=212 cm^{-1}). Similarly, whether Zn^{2+} or Cu^{2+} binding to COF (Cu^{2+}/COF, 1605 cm^{-1} and 1383 cm^{-1}, Δv = 222 cm^{-1}; Zn^{2+}/COF, 1605 cm^{-1} and 1388 cm^{-1}, Δv = 217 cm^{-1}), its interaction with the carboxyl group of PA is significantly enhanced compared with homogeneous metal ions (both Cu^{2+} and Zn^{2+} case are 1601 cm^{-1} and 1389 cm^{-1}, Δv = 212 cm^{-1}). All of our dynamic ATR-FTIR results demonstrate that the coordination between Cu^{2+}/COF or even Zn^{2+}/COF and carboxyl of both initial PG and intermediate PA is precisely conducive to decarboxylation after the main hydrolysis of β-lactam bond.

Text S12. The detailed degradation pathway of PG by Cu^{2+}/COF.

In detail, owing to the extraordinary feedback π bond in our M^{2+}/COF structure, there exists strong interaction between M^{2+} moiety of M^{2+}/COF and any suspending carboxyl groups including both nascent carboxyl formed from the β-lactam bond hydrolysis and the carboxyl on the thiazolidine ring of original PG. After excitation by light or photothermal together, high degree of charge separation makes M^{2+}/COF has a more positive charge on M^{2+} sites. This powerful polarization action has more conducive to weaken the C-COOH bond of PA, leading removing COOH and breaking C-N bond to generate PAA and 5, 5-dimethylenethiazolidine-4-carboxylic acid fragment (DMCA) by excited charge separation state (see **Figure S9-5b**). DMCA fragment is very readily decarboxylated to degradation and it is commonly difficult to detect. We detected traces of DMCA in concentrated reaction solution by LCMS (the identification result by extraction ion chromatogram and secondary mass spectrometry are shown in **Figure S9-6**). For the generation of DPDC, PA is first oxidized by the separated hole in HOMO orbit of M^{2+}/COF under light to produce imine bonds and then both carboxyl groups are removed by M^{2+}/COF activation under heating or light to produce the final product DPDC.

Text S13. The details of recycling experiments.

The reaction volume was 20 mL and the concentration of Cu^{2+}/COF was 0.2 g/L. Cu^{2+}/COF was stirred with PG solution for 30 min under darkness to reach adsorption equilibrium, then the photothermal degradation proceeded under the light. At 30 min intervals, 0.1 mL of suspension was sampled and 0.01 mL of ethylenediaminetetraacetic acid dipotassium salt (EDTA) solution (20 mM)

was spiked into samples. The mixture was shaken for 2 min to extract the adsorbed antibiotics and filtered through a 0.22 μm poly(tetrafluoroethylene) membrane. After each run, Cu^{2+}/COF was collected by filtration and put in the next run after being washed once with water.

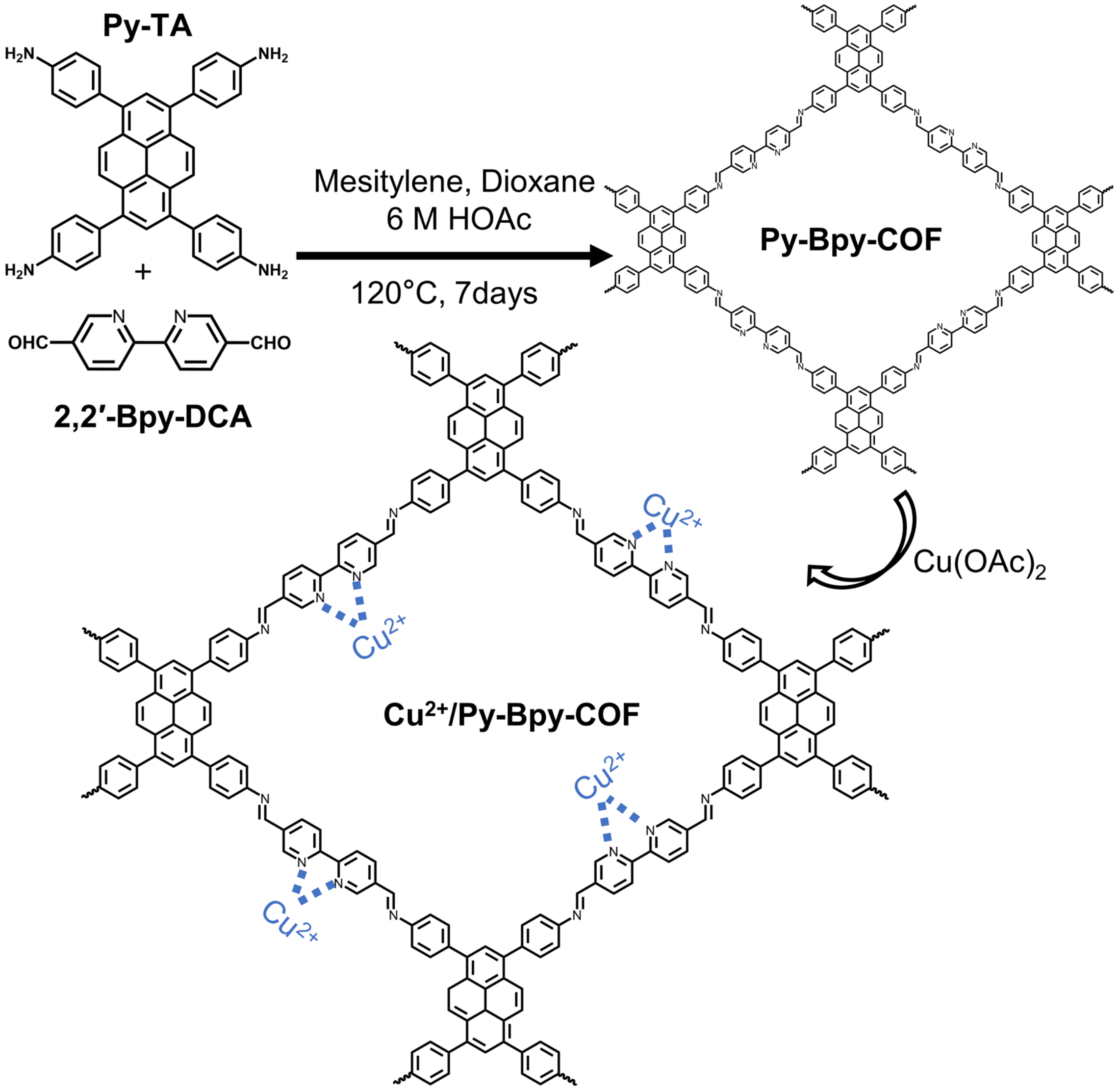

Figure S1. Schematic representation of the synthesis of Py-Bpy-COF via Schiff base condensation and Cu^{2+}/Py-Bpy-COF via Cu(II) impregnation.

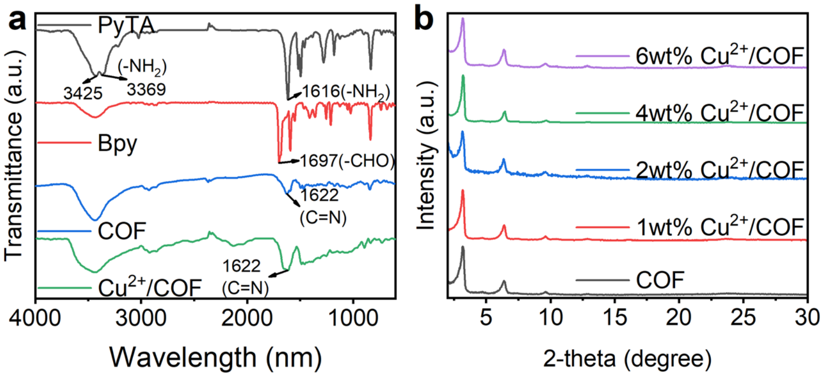

Figure S2. **(a)** FT-IR spectra of precursor PyTA, Bpy, Py-Bpy-COF and Cu^{2+}/Py-Bpy-COF. **(b)** XRD patterns of the as-prepared COF and different content Cu^{2+}/COF.

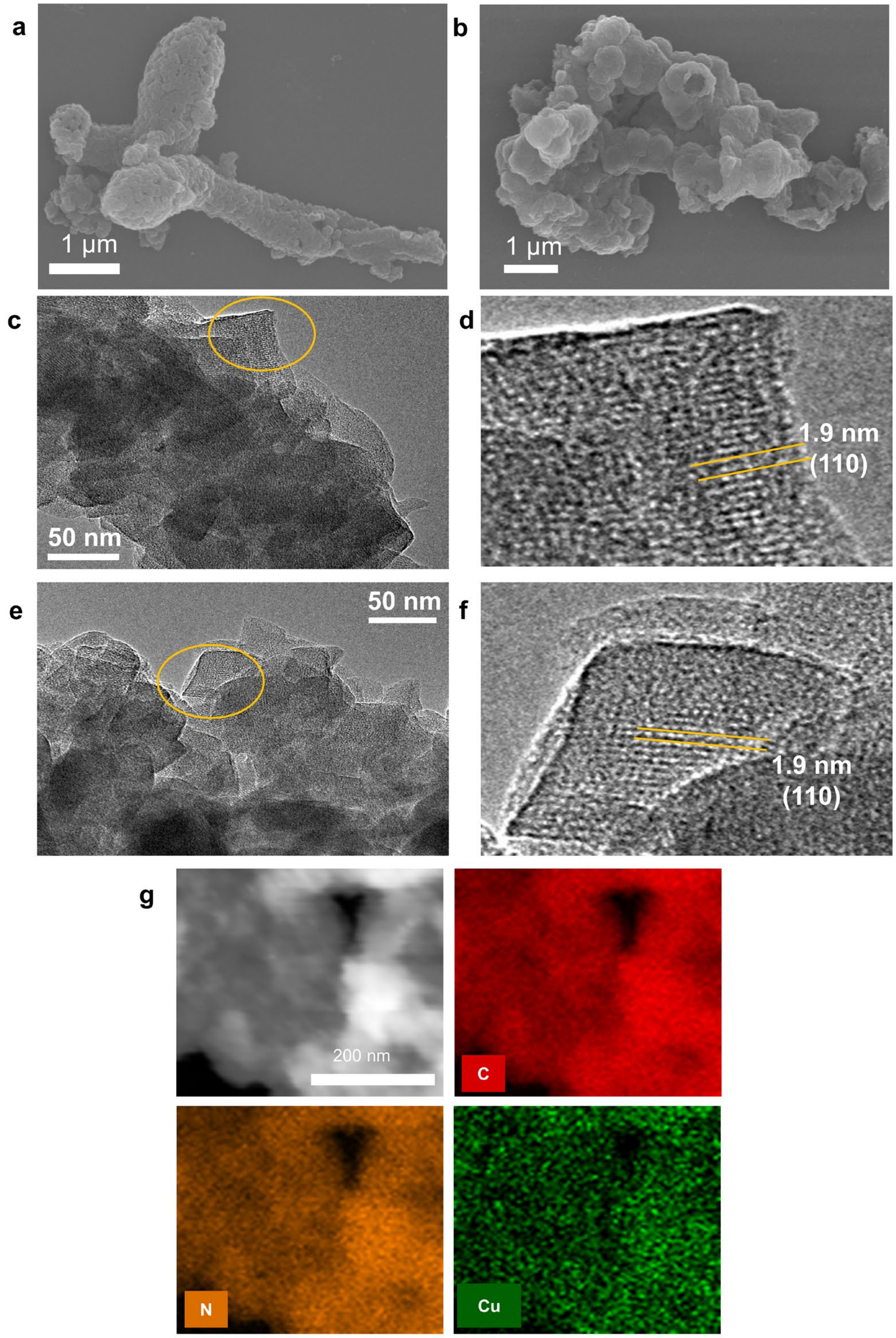

Figure S3-1. SEM image of COF **(a)** and Cu^{2+}/COF **(b)**. TEM image and partially enlarged view of COF **(c, d)** and Cu^{2+}/COF **(e, f)**. The labels in **d** and **f** are lattice spacing. **(g)** HAADF−STEM image and the corresponding EDS elemental mapping of Cu^{2+}/COF.

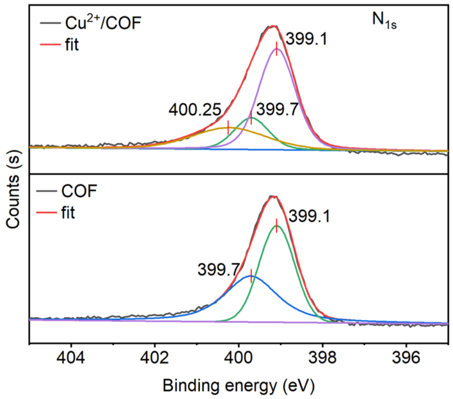

Figure S3-2. XPS spectra of COF and Cu^{2+}/COF.

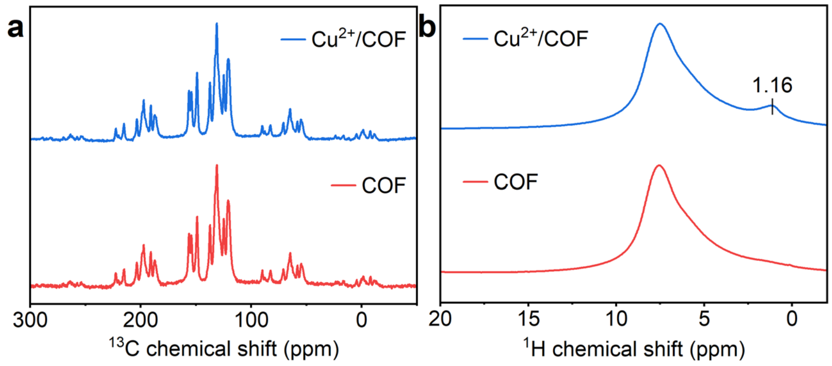

Figure S3-3. The solid-state ^{13}C **(a)** and ^{1}H **(b)** NMR spectra of COF and Cu^{2+}/COF.

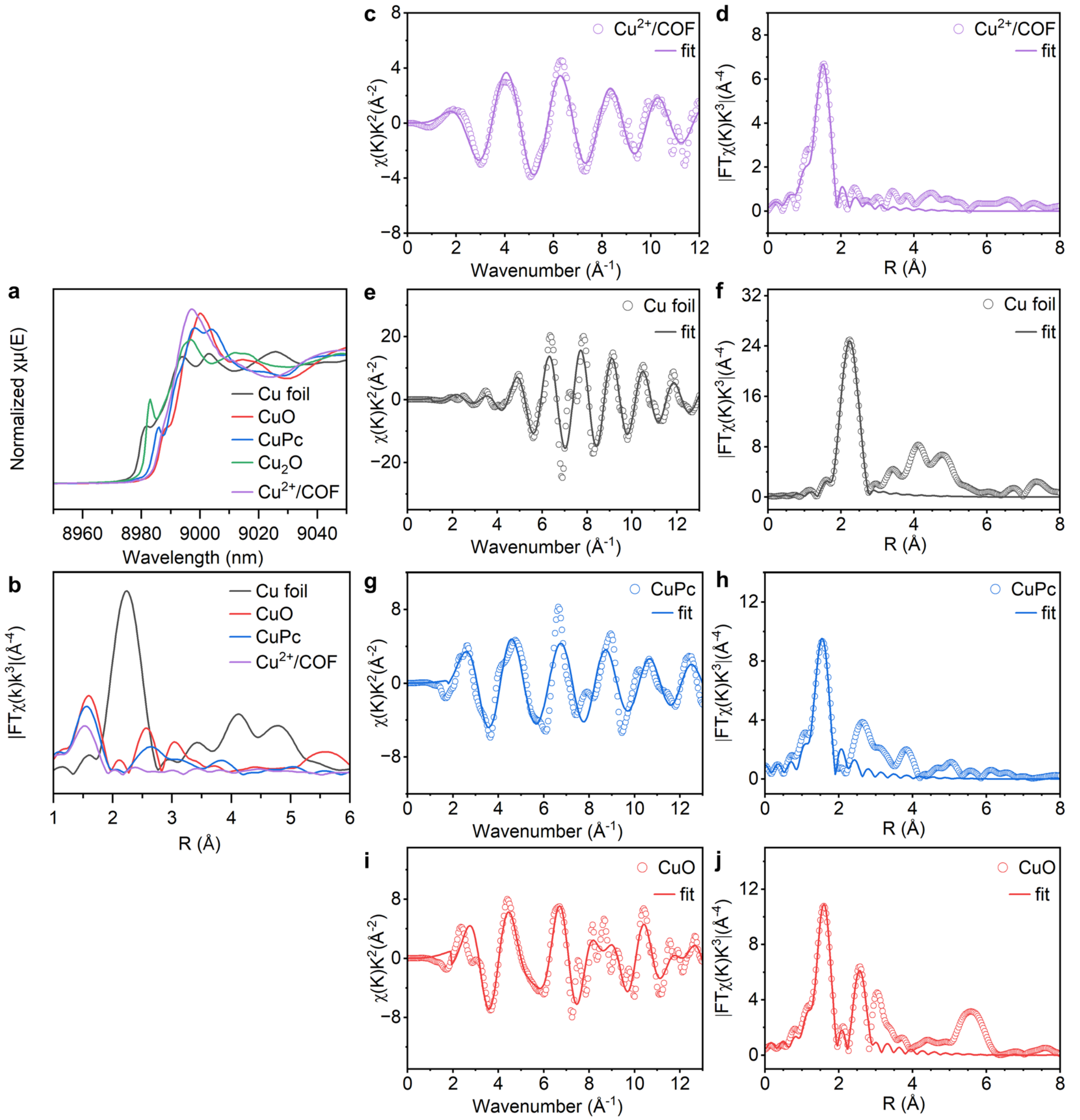

Figure S3-4. **(a)** Copper K-edge XANES spectra for Cu^{2+}/COF, CuPc, Cu_2O, CuO, and Cu foil, **(b)** Fourier transformed k^3-weighted $\chi(k)$ function of EXAFS for Cu^{2+}/COF, CuPc, CuO, and Cu foil. Fitting of the EXAFS spectrum of Cu^{2+}/COF **(c, d)**, Cu foil **(e, f)**, CuPc **(g, h)** and CuO **(i, j)** in k space and R space.

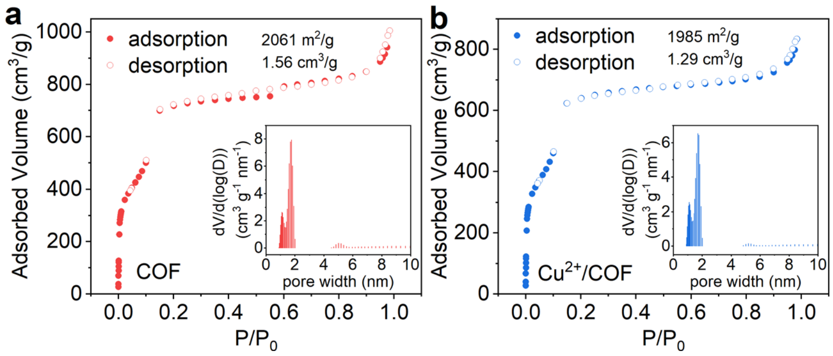

Figure S3-5. Nitrogen sorption isotherms of COF **(a)** and Cu^{2+}/COF **(b)** measured at 77 K. The inset plot is a pore-size distribution.

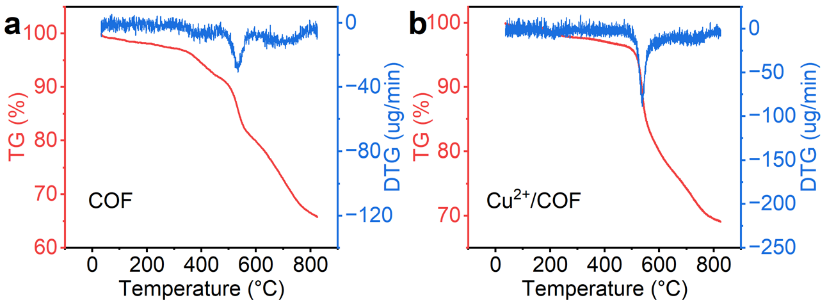

Figure S3-6. Thermogravimetric analysis of COF **(a)** and Cu^{2+}/COF **(b)**.

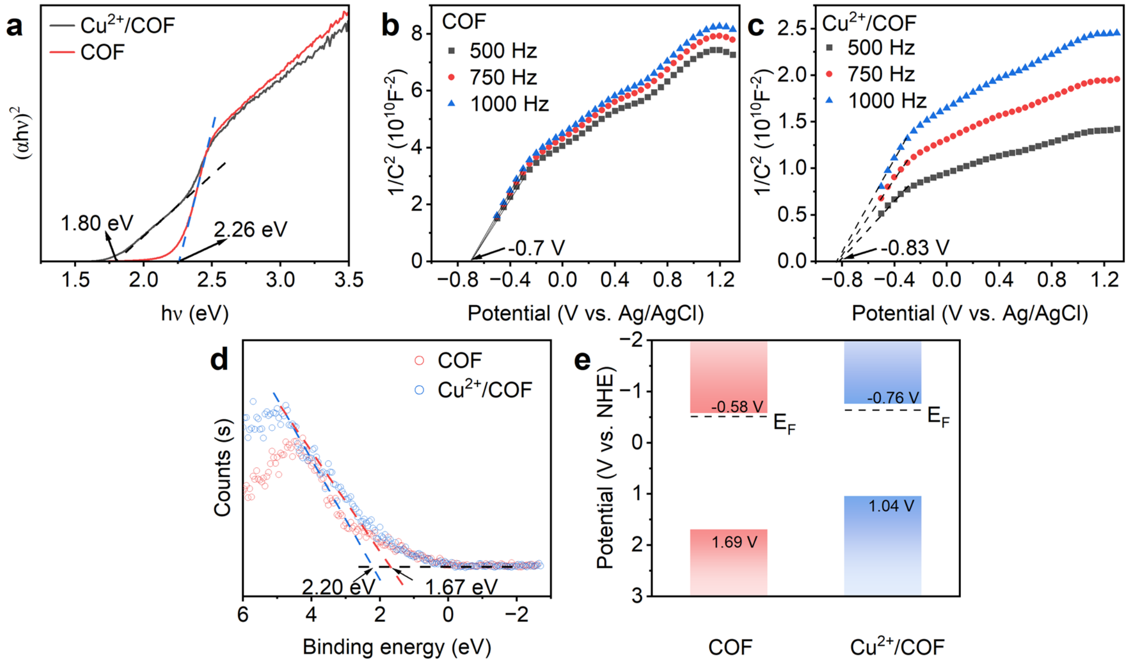

Figure S4. Tauc plots **(a)**, Mott-Schottky plots **(b, c)**, VB-XPS spectrum **(d)** and band structure **(e)** of COF and Cu^{2+}/COF.

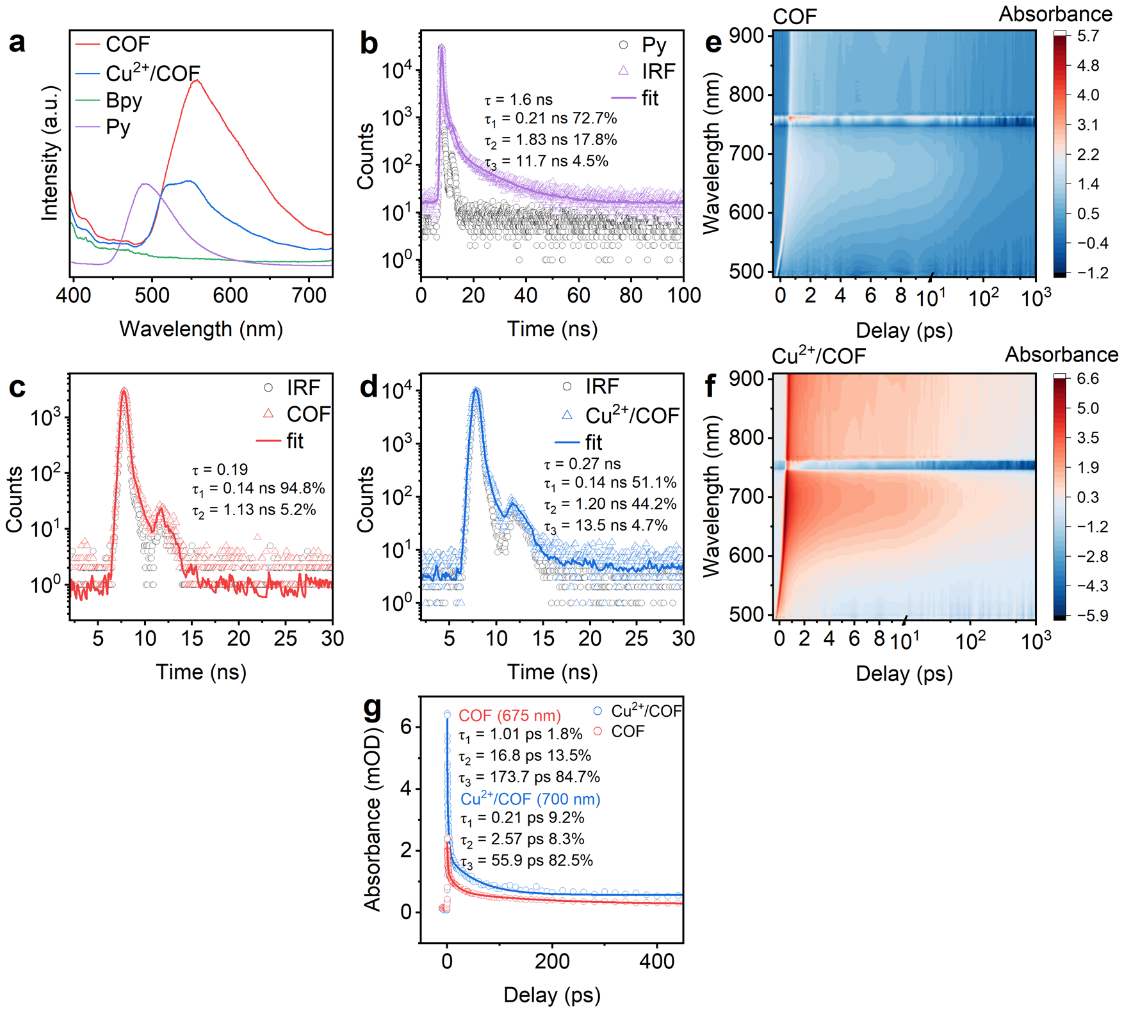

Figure S5-1. **(a)** The fluorescence spectra of COF, Cu^{2+}/COF, Bpy and Py. Fluorescence lifetime decay of Py **(b)** at 490 nm, COF **(c)** at 555 nm and Cu^{2+}/COF **(d)** at 550 nm. 2D femtosecond transient absorption (TA) spectra of COF **(e)** and Cu^{2+}/COF **(f)**. **(g)** The TA kinetic curves and fitting results for COF (probed at 675 nm) and Cu^{2+}/COF (probed at 700 nm). The excitation wavelength of the above measurements is all 375 nm.

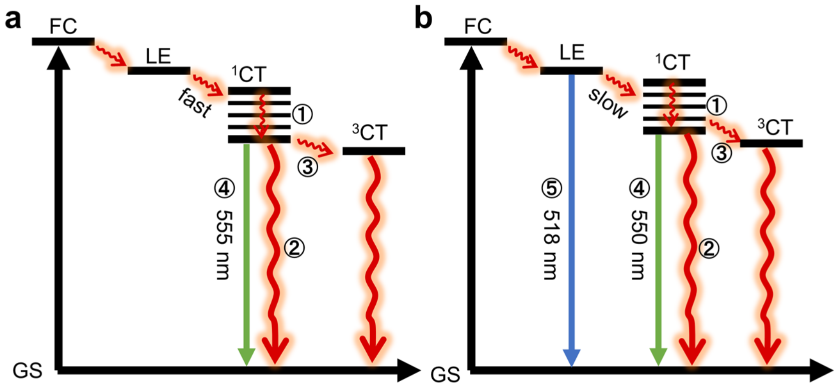

Figure S5-2. The proposed Jablonski diagram of COF **(a)** and Cu^{2+}/COF **(b)**. (GS, ground state; FC, Frank–Condon state; LE, locally excited state; ^{1}CT, charge transfer singlet state; ^{3}CT, charge transfer triplet state; ①vibration relaxation; ②internal conversion (IC); ③intersystem crossing (ISC); ④⑤fluorescence)

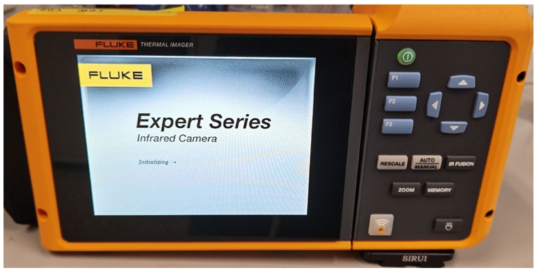

Figure S6. The digital photo of the infrared thermal camera.

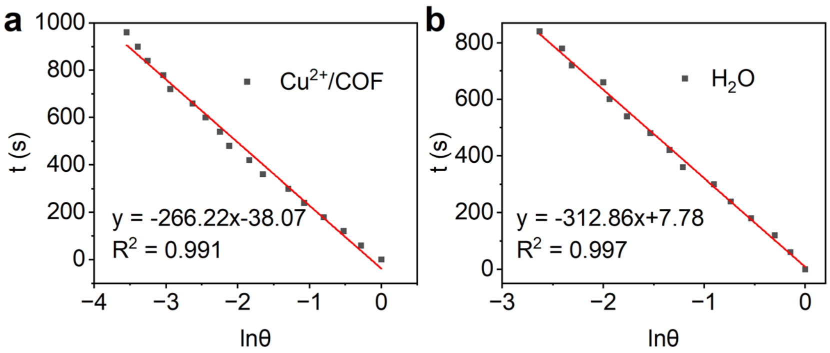

Figure S7. Linear time data versus −ln θ obtained from the cooling period of **Figure 2h**.

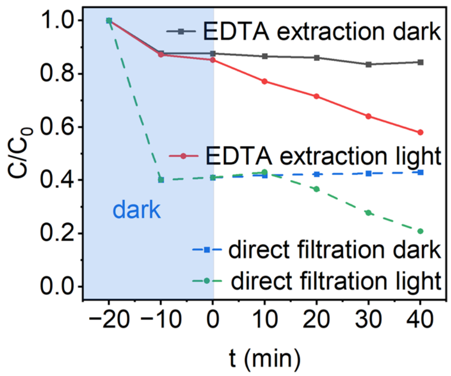

Figure S8-1. PG degradation test with direct filtration and EDTA extraction.

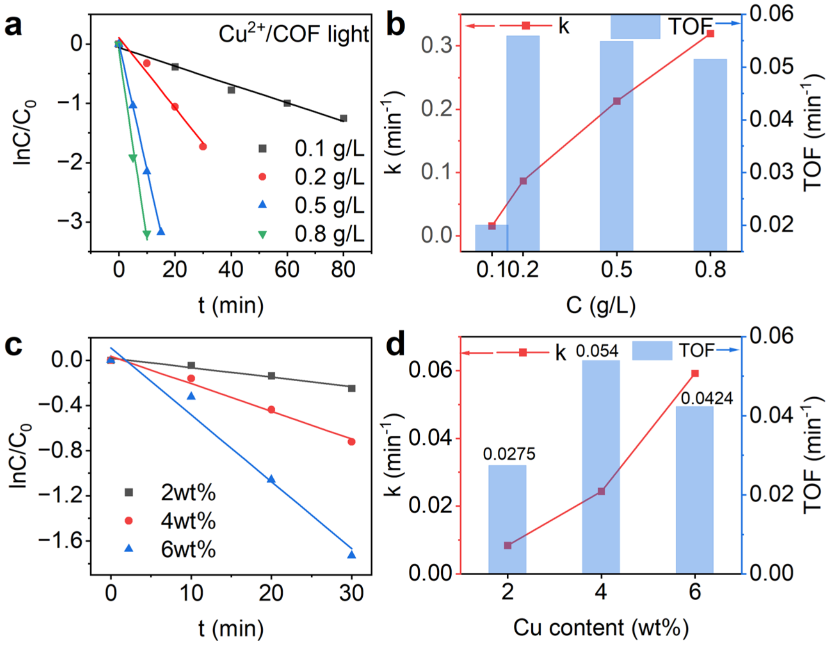

Figure S8-2. **(a)** PG degradation kinetic and **(b)** degradation rate constant or TOF with different concentrations of Cu^{2+}/COF (8 wt%) under illumination. **(c)** PG degradation kinetic and **(d)** degradation rate constant or TOF with different content of Cu^{2+}/COF but same concentration (0.2 g/L) under illumination.

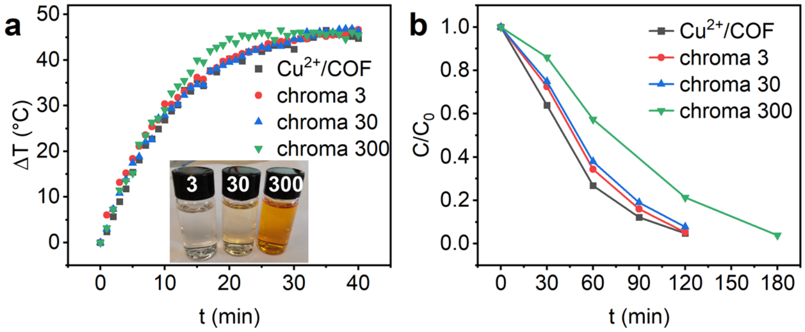

Figure S8-3. **(a)** The temperature variation curve (inset is the picture of different chroma of solution) and **(b)** PG degradation performance of Cu^{2+}/COF dispersion with different chroma under illumination.

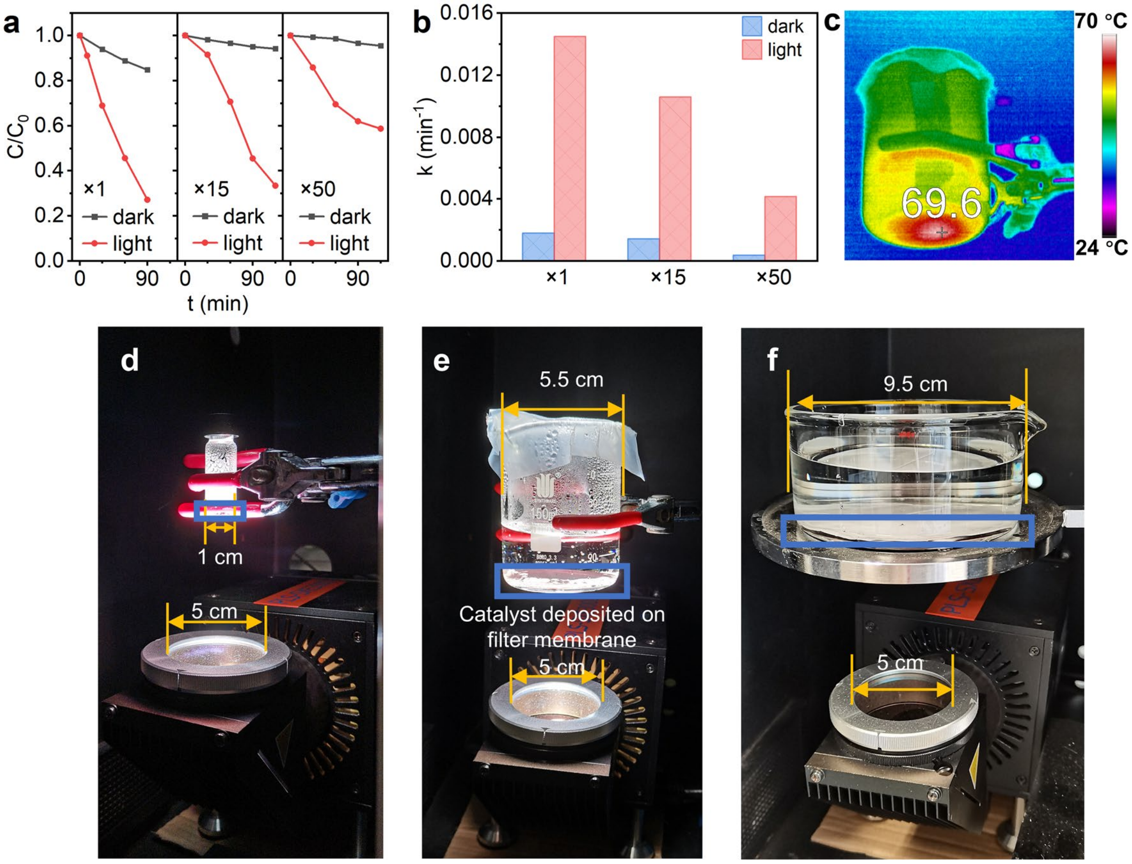

Figure S8-4. **(a)** The PG degradation performance and **(b)** rate constant of Cu^{2+}/COF under local photothermal and dark conditions with the reaction system were magnified 15 times (×15) and 50 times (×50). **(c)** The infrared thermal image of the reaction system was magnified 15 times. **(d)** The local photothermal PG degradation equipment (ordinary) and equipment of the reaction system magnified to 15 times **(e)** and 50 times **(f)**.

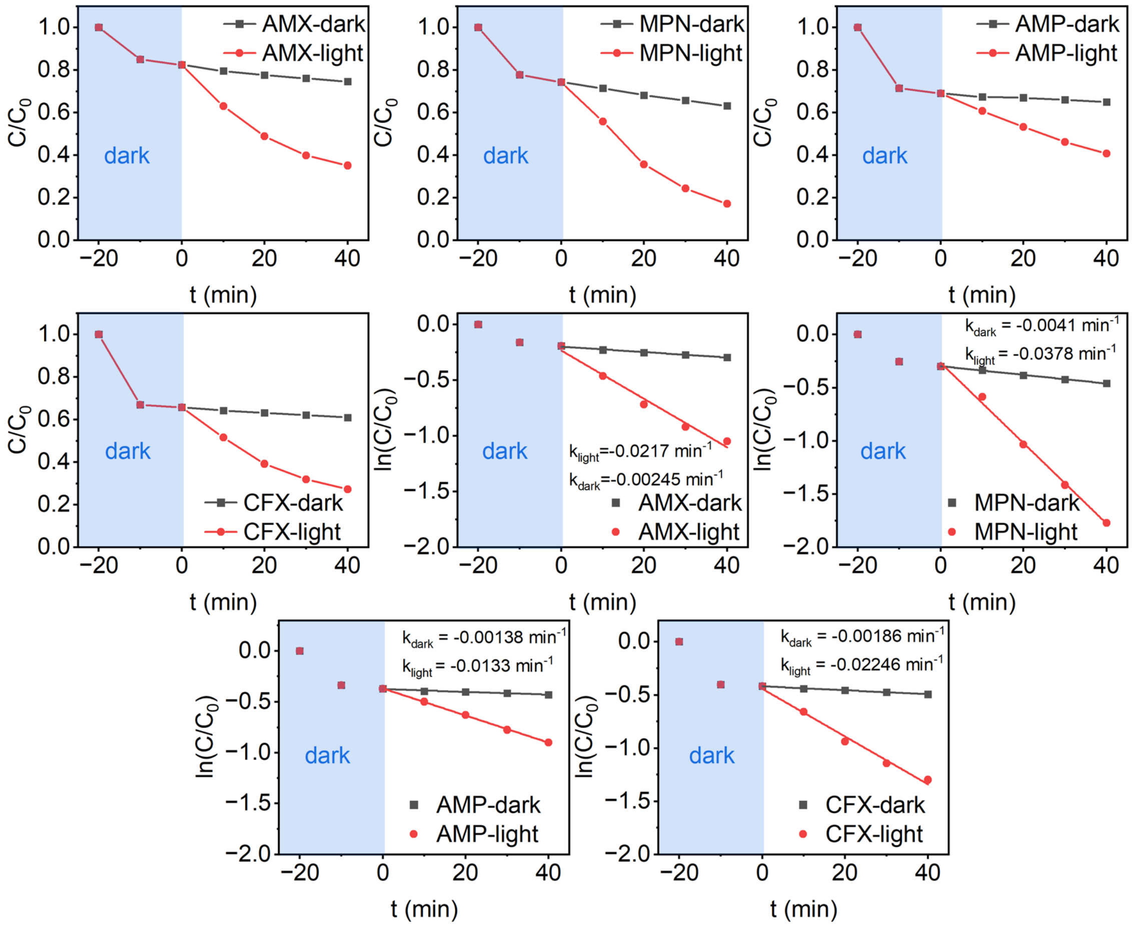

Figure S8-5. The degradation rate and kinetics of various β-lactam antibiotics (C_0 = 0.1 mM) such as MPN, CFX, AMP and AMX with Cu^{2+}/COF under dark and light conditions.

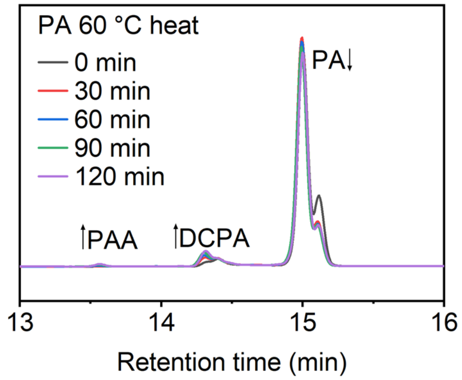

Figure S9-1. The LC spectra showed the decomposition of the PA solution with the heating time (at 60 °C).

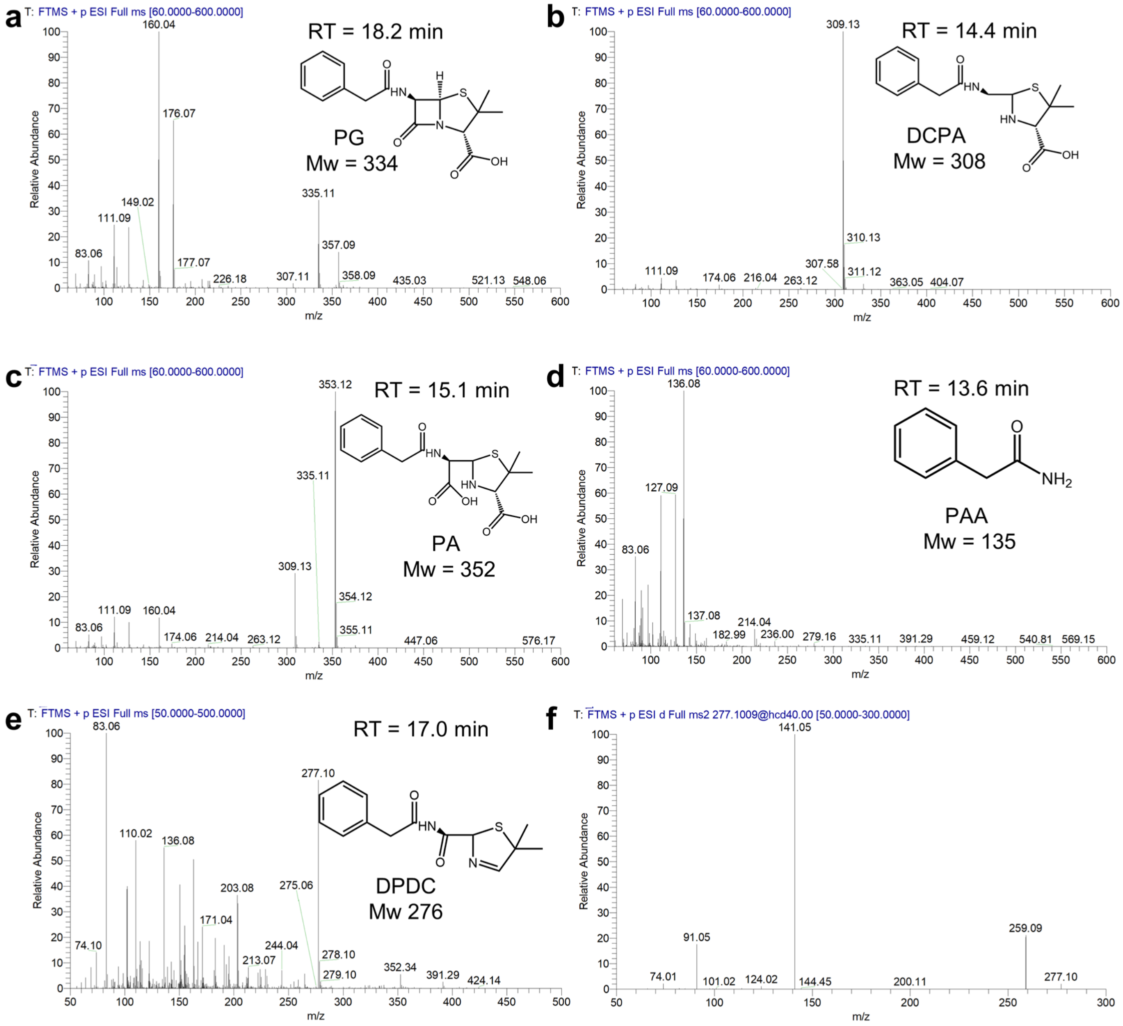

Figure S9-2. The full scan MS1 of corresponding retention time (13.6 min, 14.4 min, 15.1 min, 17.0 min and 18.2 min) and MS2 of 17.0 min detected by LC-MS.

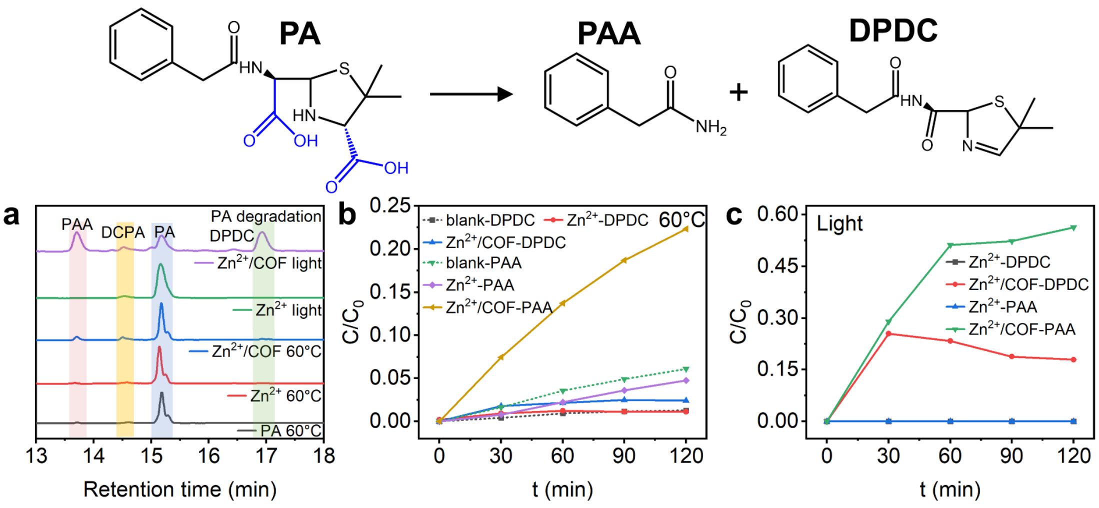

Figure S9-3. **(a)** LC spectra showed the PA degradation products mediated by Zn^{2+}/COF and Zn^{2+} under heat (60 °C) and light conditions for 30 min. **(b)** The change of the production amount of PAA and DPDC of PA degradation by Zn^{2+}/COF, Zn^{2+} and no catalyst (blank) after heat (60 °C). **(c)** The change of the production amount of PAA and DPDC of PA degradation by Zn^{2+}/COF and Zn^{2+} under light condition.

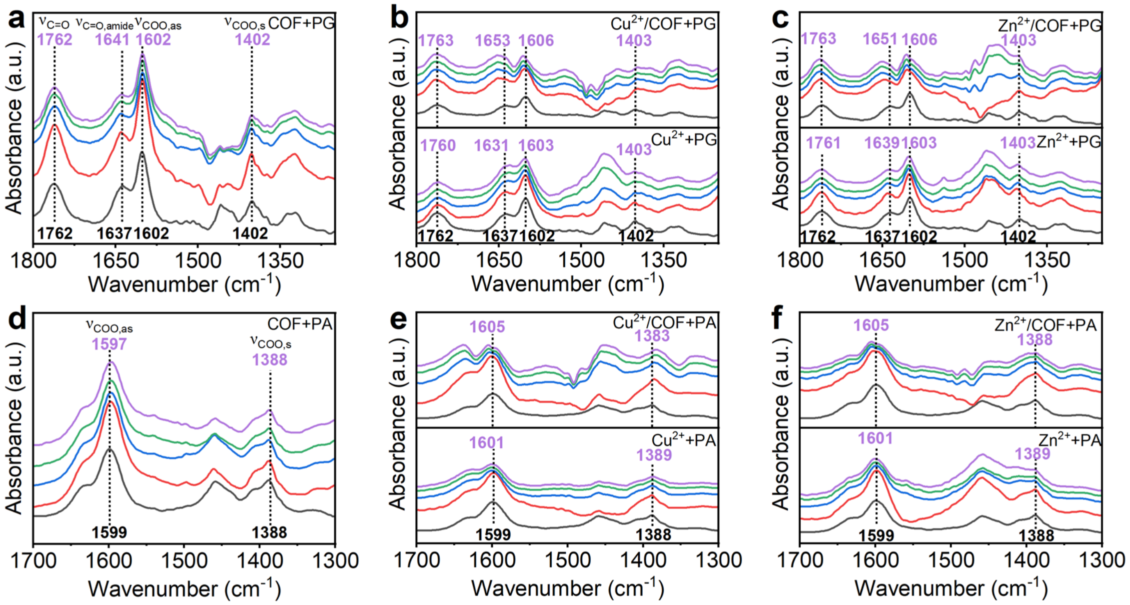

Figure S9-4. ATR−FTIR spectra of PG with **(a)** COF dispersion, **(b)** Cu^{2+} solution (bottom) and Cu^{2+}/COF dispersion (top), **(c)** Zn^{2+} solution (bottom) and Zn^{2+}/COF dispersion (top). ATR−FTIR spectra of PA in **(d)** COF dispersion, **(e)** Cu^{2+} solution (bottom) and Cu^{2+}/COF dispersion (top), **(f)** Zn^{2+} solution (bottom) and Zn^{2+}/COF dispersion (top). The grey line was IR of PG/PA, the red, green, blue and purple lines are the IR spectra of PG/PA combined with the catalyst for 1 min, 5 min, 10min and 15 min respectively.

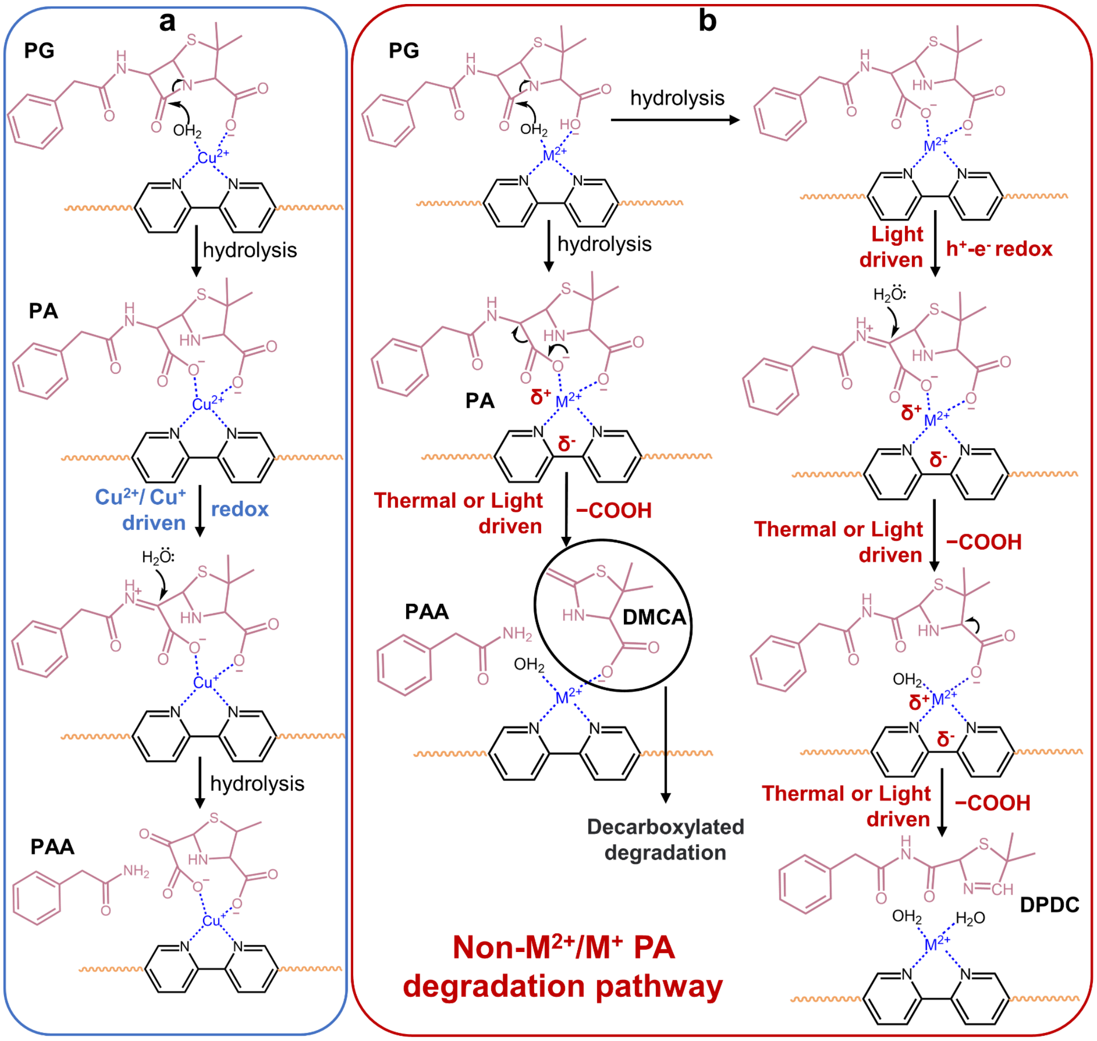

Figure S9-5. The further degradation pathway of PG after hydrolysis with M^{2+}/COF through **(a)** the reported Cu^{2+}/Cu^{+} cycle pathway (left, blue box) and **(b)** our proposed non-M^{2+}/M^{+} cycle pathway (right, red box).

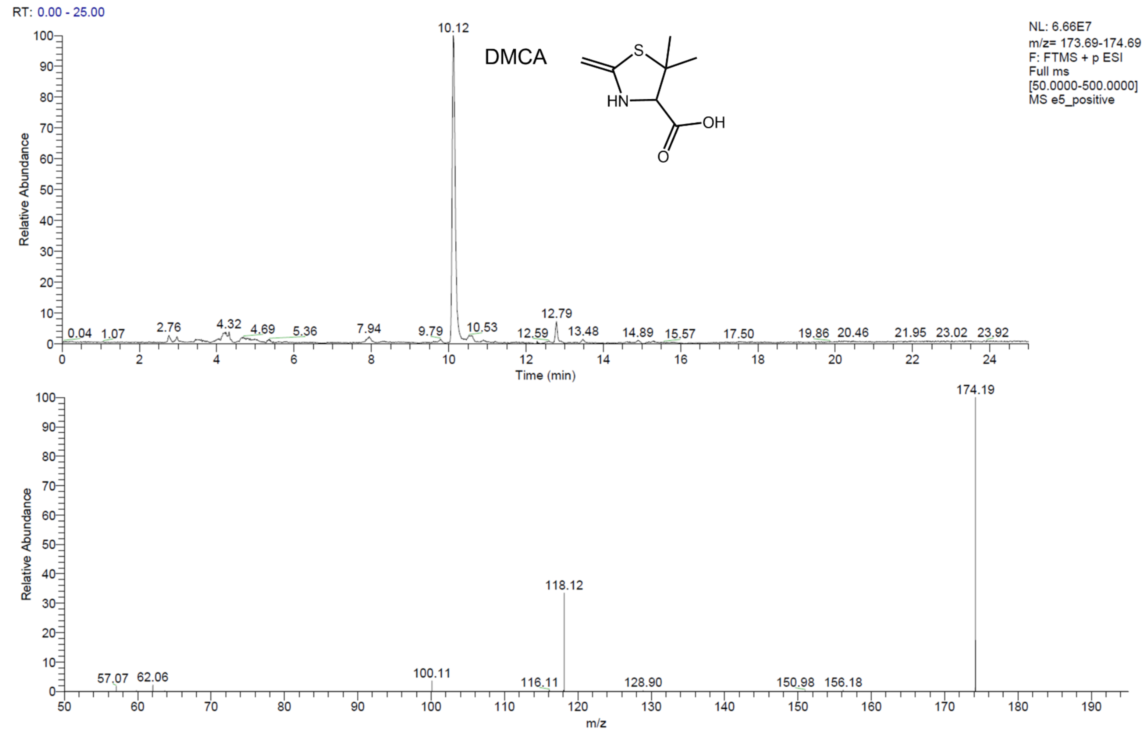

Figure S9-6. The extraction ion chromatogram and secondary mass spectrometry of DMCA.

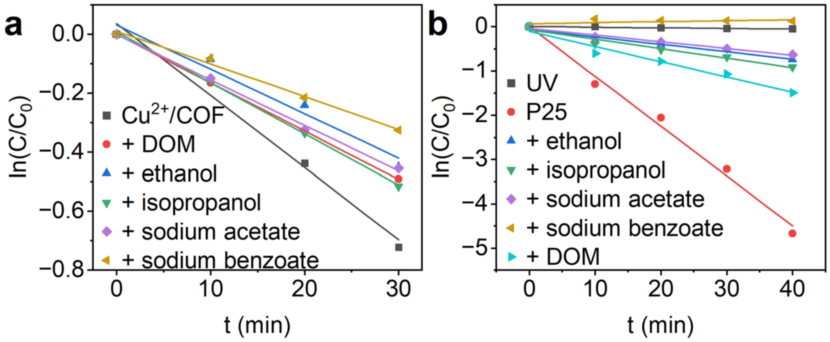

Figure S10. The PG degradation kinetics of **(a)** Cu^{2+}/COF under the photothermal condition and **(b)** P25-TiO_2 under the UV photocatalytic condition with different interference.

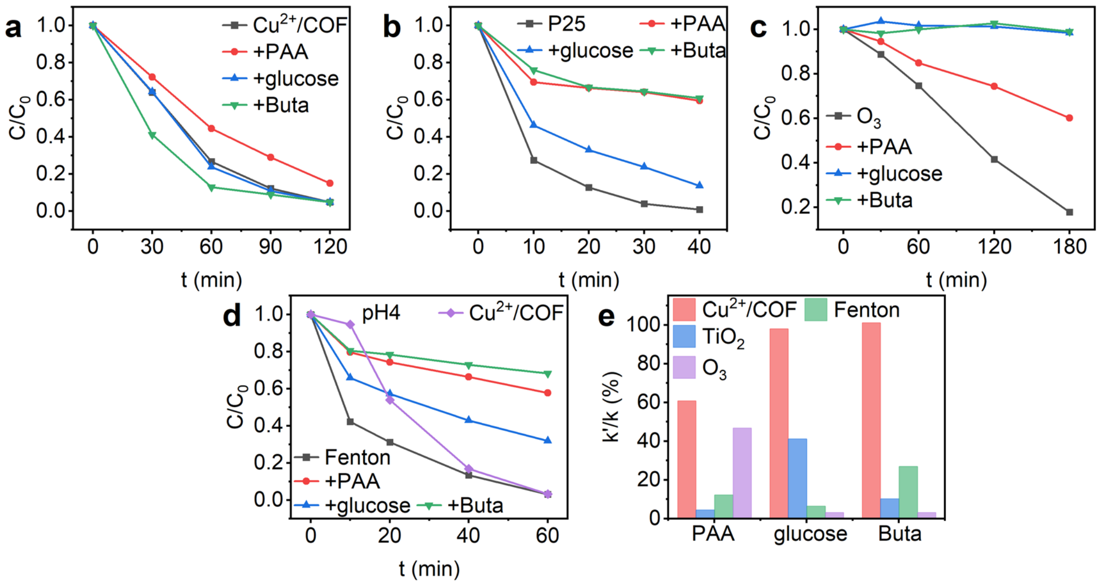

Figure S11. The PG (C_0 = 0.1 mM) degradation performance of **(a)** Cu^{2+}/COF photothermal hydrolysis (Cu^{2+}/COF: 0.2 g/L, simulated sunlight, 1 W/cm^2), **(b)** P25 photocatalysis (P25: 0.2 g/L, UV light, 100 mW/cm^2), **(c)** O_3 oxidation (30 ppm, 30 mL/min) and **(d)** Fenton (pH4, $[Fe^{2+}]$ = 0.045 mM, $[H_2O_2]_0$ = 0.9 mM) with phenylacetamide (PAA, 0.5 g/L), butyl acetate (Buta, 10 g/L) and glucose (0.1 g/L). **(e)** The ratio of rate constant with interferent (noted as k') to without interferent (noted as k) among Cu^{2+}/COF photothermw2al degradation, P25 photocatalysis, O_3 oxidation and Fenton technology.

Table S1. HPLC method parameters for detection of β-lactam antibiotics.

Chemical	Mobile Phase	Wavelength (nm)	Flow Rate (mL/min)
PG	water with 0.1 % H_3PO_4/acetonitrile (35:65 v/v)	210	0.4
AMX	water with 0.1 % H_3PO_4/methanol (80:20 v/v)	230	0.8
AMP	water with 0.1 % H_3PO_4/methanol (65:35 v/v)	230	0.8
CFX	water with 0.1 % H_3PO_4/methanol (70:30 v/v)	262	0.8
MPN	water with 0.1 % H_3PO_4/methanol (80:20 v/v)	296	0.8

Table S2. ICP-MS results of different content Cu^{2+}/COF.

Theoretical (wt%)	1	2	4	6	8
Measured (wt%)	0.65	0.97	1.43	4.44	4.91

Table S3. EXAFS fitting parameters at the Cu K-edge for Cu foil, CuO, CuPc and Cu^{2+}/COF.

Sample	Shell	CN[a]	R (Å)[b]	σ^2 (10^{-3}Å^2)[c]	ΔE_0 (eV)[d]	R factor
Cu foil	Cu-Cu	12*	2.54±0.003	8.58±0.17	4.26±0.52	0.003
CuO	Cu-O	4.0*	1.94±0.007	4.10±0.78	14.23±0.64	0.013
	Cu-O	2.0*	2.86±0.000	0.10±0.00		
	Cu-Cu	2.0*	2.90±0.000	11.70±3.62		
CuPc	Cu-N	4.7±1.0	1.93±0.017	3.56±2.27	11.69±2.47	0.020
Cu^{2+}/COF	Cu-N/O	3.9±0.4	1.94±0.008	5.52±1.23	-2.75±1.18	0.013

[a]*CN*, coordination number; [b]*R*, distance between absorber and backscatter atoms; [c]σ^2, Debye-Waller factor to account for both thermal and structural disorders; [d]ΔE_0, inner potential correction; *R* factor indicates the goodness of the fit. S_0^2 was fixed to 0.85. A reasonable range of EXAFS fitting parameters: 0.600 < S_0^2 < 1.000; *CN* > 0; σ^2 > 0 Å^2; |ΔE_0| < 15 eV; *R* factor < 0.02. Fitting range: 3.0 ≤ k (/Å) ≤ 12.0 and 1.0≤ R (Å) ≤ 3.0 (Cu foil); 3.0 ≤ k (/Å) ≤ 13.0and 1.0 ≤ R (Å) ≤ 2.8 (CuO); 3.0≤k (/Å) ≤ 12.0 and 1.0 ≤ R (Å) ≤ 2.0(CuPc); 3.0≤k (/Å) ≤ 12.0 and 1.0 ≤ R (Å) ≤ 2.0(Cu sample)

SI References

1. B. Ravel, M. Newville, ATHENA and ARTEMIS: interactive graphical data analysis using IFEFFIT. *Phys. Scr.* **2005**, 1007 (2005).
2. X. Chen *et al.*, Towards covalent organic frameworks with predesignable and aligned open docking sites. *Chem. Commun.* **50**, 6161-6163 (2014).
3. H. B. Aiyappa, J. Thote, D. B. Shinde, R. Banerjee, S. Kurungot, Cobalt-modified Covalent Organic Framework as a robust water oxidation electrocatalyst. *Chem. Mater.* **28**, 4375-4379 (2016).
4. X. Li *et al.*, Facile transformation of imine covalent organic frameworks into ultrastable crystalline porous aromatic frameworks. *Nat. Commun* **9**, 2998 (2018).
